# Mediterranean Plants as Potential Source of Biopesticides: An Overview of Current Research and Future Trends

**DOI:** 10.3390/metabo13090967

**Published:** 2023-08-22

**Authors:** Regina Fragkouli, Maria Antonopoulou, Elias Asimakis, Alexandra Spyrou, Chariklia Kosma, Anastasios Zotos, George Tsiamis, Angelos Patakas, Vassilios Triantafyllidis

**Affiliations:** 1 Department of Food Science & Technology, University of Patras, Seferi 2, 30100 Agrinio, Greece; rfragkouli@upatras.gr (R.F.); xkosma@upatras.gr (C.K.); apatakas@upatras.gr (A.P.); 2 Department of Sustainable Agriculture, University of Patras, Seferi 2, 30100 Agrinio, Greece; mantonop@upatras.gr (M.A.); eliasasim@upatras.gr (E.A.); up1096213@upatras.gr (A.S.); azotos@upatras.gr (A.Z.); gtsiamis@upatras.gr (G.T.)

**Keywords:** biopesticides, plant extracts, essential oils, extraction methods, chemical composition, antimicrobial activity, insecticidal activity, herbicidal activity, alternative agriculture

## Abstract

The development and implementation of safe natural alternatives to synthetic pesticides are urgent needs that will provide ecological solutions for the control of plant diseases, bacteria, viruses, nematodes, pests, and weeds to ensure the economic stability of farmers and food security, as well as protection of the environment and human health. Unambiguously, production of botanical pesticides will allow for the sustainable and efficient use of natural resources and finally decrease the use of chemical inputs and burden. This is further underlined by the strict regulations on pesticide residues in agricultural products and is in harmony with the Farm to Fork strategy, which aims to reduce pesticide use by 50% by 2030. Thus, the present work aims to compile the scientific knowledge of the last 5 years (2017–February 2023) regarding the Mediterranean plants that present biopesticidal effects. The literature review revealed 40 families of Mediterranean plants with at least one species that have been investigated as potential biopesticides. However, only six families had the highest number of species, and they were reviewed comprehensively in this study. Following a systematic approach, the extraction methods, chemical composition, biopesticidal activity, and commonly used assays for evaluating the antimicrobial, pesticidal, repellant, and herbicidal activity of plant extracts, as well as the toxicological and safety aspects of biopesticide formulation, are discussed in detail. Finally, the aspects that have not yet been investigated or are under-investigated and future perspectives are highlighted.

## 1. Introduction

Climate change and environmental degradation are severe threats worldwide, and their consequences can cause serious impacts on our planet. Recognizing the importance of these threats to humanity, on 11 December 2019, the EU Commission presented the European Green Deal, which consists of a set of policy initiatives that aim to neutralize climate by 2030 and render Europe the first climate-neutral continent by 2050 [1]. One of these initiatives is the reduction of greenhouse gas emissions by at least 55% by 2030 compared to 1990 levels. To achieve 2030 climate targets, the EU Commission has also adopted a set of strategies in various sectors such as transportation, industry, energy, and agriculture [2].

Amongst them, the Farm to Fork strategy is characterized as the heart of the European Green Deal and aims to accelerate the transition to a sustainable food system. The objective of this strategy is to ensure food safety in an environmentally sustainable manner, simultaneously maximizing environmental, health, and social benefits. To accelerate the transition to sustainable and healthy food systems, this strategy aims to reduce pesticide use by 50% by 2030 by applying low-input sustainable agriculture or simply alternative agriculture, amongst others [2].

Pesticides are any substance or mixture of substances of chemical or biological ingredients intended for repelling, destroying, or controlling any pest or for regulating plant growth [3]. The term “pesticide” applies to insecticides, herbicides, fungicides, rodenticides, molluscicides, wood preservatives, and various other substances used to control pests. Pesticides also include plant growth regulators, defoliants, and desiccants. Their use has increased 50% since 1950, and it is estimated that 2.5 million tons of industrial pesticides are now used each year [4]. Moreover, global pesticide use is expected to show an increasing trend in the future, and it is expected to reach a value of 4.5 million tons by 2030 [5,6].

Although pesticides have a principal role in crop production, intensive and improper use of them can cause numerous detrimental effects on human health and the environment and reduce the safety of agricultural products, which has raised major public and scientific concern in the last few decades [7,8,9]. For humans, dermatological, gastrointestinal, neurological, carcinogenic, respiratory, reproductive, and endocrine effects are representative adverse health effects that have been associated with pesticide exposure [10].

The human and environmental health risks that are associated with the use of chemical pesticides, as well as the aims set by the Farm to Fork strategy, have led to an increasing demand for the development of alternative eco-friendly pesticide formulations. Biopesticides have long been recognized as attractive alternatives to synthetic chemical pesticides for pest control because they present important properties, with their non-toxic nature being the most significant [11,12,13].

Biopesticides aim to control plant-damaging pests, insects, and fungi and are generally categorized into three groups: (i) microbial biopesticides (containing microorganisms like bacteria, fungi, viruses, and protozoan or entomopathogenic nematodes as active ingredients that attack specific pest species), (ii) biochemical biopesticides (containing naturally occurring substances that control pests via non-toxic mechanisms), and (iii) plant-incorporated protectants (containing substances produced by plants from genetic material that has been added to the plant) [11,12]. The practice of using plant derivatives in agriculture has a long history of at least two and a half millennia, dating back to ancient Greece and Rome [14]. Botanical pesticides are characterized by bioactive mixtures/extracts/compounds from plant materials that serve as insecticides and repellents but also as bactericides, fungicides, herbicides, and nematicides [15]. In general, botanical pesticides contain numerous compounds that can be volatile and belong to different chemical groups such as aldehydes, ketones, alcohols, heterocycles, ethers or oxides, phenols, esters, amines, amides, flavonoids, and terpenes, amongst others. All of these compounds are produced as secondary metabolites and can present activities against pests, insects, and pathogenic fungi. Representative examples are the well-documented antimicrobial and antioxidant properties that present various terpenoids and phenolic compounds [13]. However, few biopesticide formulations have been commercialized up to now. The main limitations concern their reduced storage stability and sensitivity to environmental conditions, as well as the high production cost, which should be overcome in the near future. In this direction, the improvement of the formulation to increase and maintain the activity of biopesticides could be a solution [13]. Moreover, the use of widely available plants as raw materials can also contribute to overcoming the existing limitations.

As plant-based natural pesticides have gained considerable attention in the few last years and development of them is still a growing trend, there is an urgent need to compile the current scientific knowledge about plants presenting biopesticidal effects, especially for the countries where the source plants are readily available and where conventional formulations comprising synthetic pesticides are both expensive and dangerous to humans and the environment. Being aware of the above, numerous researchers have focused on the evaluation of extracts and essential oils with biopesticidal properties from plants of Mediterranean countries. Therefore, this study provides an overview of the current research on botanical pesticides native to Mediterranean countries for the period of 2017–February 2023.

Based on the overview, the extraction methods, chemical composition, biopesticide activity, and commonly used assays for evaluating the antimicrobial, pesticidal, repellant, and herbicidal activity of plant extracts are discussed. Special attention is also devoted to toxicological and safety aspects that should be considered before the commercialization of biopesticide formulations. Finally, the gaps in the literature that should be investigated and future perspectives are highlighted.

## 2. Mediterranean Plants That Have Been Recently Investigated for Biopesticidal Activity

The literature review between 2017 and February 2023 revealed 40 families with at least one species of scientific interest as potential biopesticides (Table 1). Among them, six families had the highest number of species and are presented in detail below. It is worth mentioning the existence of other families like Meliaceae and Rutaceae, which are of great scientific interest, but as scientific articles focused on these species native to Mediterranean countries were not published in the examined period, these families are not analyzed below. The biological activity of the species (and generally of the families) is determined by the chemical composition of the secondary metabolites. According to Pichersky and Gang [16], secondary metabolites are compounds whose biosynthesis is restricted to selected plant groups and serve specific needs of the plant (e.g., insect attraction, resistance to salt or drought).

### 2.1. Lamiaceae

Lamiaceae (or Labiatae) is a family of plants composed of 7530 species [17] (trees, shrubs, subshrubs, and herbs) that are characterized by annual or perennial carriage [18,19]. It can be found all over the planet and has several species of aromatic plants that are used in medicine, in the pharmaceutical and food industries [20], and as ornamental plants. The most interesting species, with several biological applications, belong to the genera *Thymus* (e.g., *Thymus vulgaris*), *Origanum* (e.g., *Origanum vulgare*), *Salvia* (e.g., *Salvia rosmarinus*) and common garden sage (e.g., *Salvia officinalis*), *Melissa* (e.g., *Melissa officinalis*)*, Levandula* (e.g., *Lavandula angustifolia*), *Mentha* (e.g., *Mentha spicata*), and *Ocimum* (e.g., *Ocimum basilicum*) [21]. Essential oils of these species have been reported to possess strong insecticidal, acaricidal, fungicidal, and herbicidal activity, in addition to other biological activity such as antioxidant, antitumor, anti-inflammatory, antiviral, analgesic, antitussive, antiasthmatic, and antimicrobial activitiy [22,23,24].

All of this activity is determined by the chemical composition of the essential oils. In general, the species of Lamiaceae produce large amounts of secondary metabolites and, based on the volatility of the compounds, they can be distinguished into two groups:

- Species that mainly produce volatile terpenoids in their essential oils;
- Species that mainly produce nonvolatile metabolites and poor essential oils [19].

According to Table 1, Lamiaceae species are especially rich in monoterpenes and sesquiterpenes, as they were found to be frequent constituents of Lamiaceae essential oils. More specifically, the essential oils are characterized by large quantities of some well-known compounds, like carvacrol (*Origanum*, *Satureja*, and *Thymus* species), camphor (*Lavandula* species and *S. rosmarinus*), menthol (*Mentha* species), and thymol (*Origanum* and *Thymus* species), that can present biological activities individually or synergistically with other compounds [25,26]. In general, the chemical composition of essential oils is affected by several factors, such as species, seasonality, plant age, and geographic location, as well as the extraction method [27]. For example, the composition of the essential oil of *Thymus vulgaris* varies both qualitatively and quantitively among plants collected from different geographical locations (Spain, Serbia, and Tunisia) and was investigated by Valcárcel et al. [28], Sarić-Krsmanović et al. [29], and Ben Jabeur et al. [30]. 

### 2.2. Asteraceae

Asteraceae (or Compositae) is the largest family of plants in the Angiosperms [31]. It is represented by more than 24,000 described species, which constitute 10% of all flowering species [32] and are characterized by annual or perennial carriage. Most of the species are herbaceous, and only a small number are shrubs and trees [33]. It includes crops with nutritional (lettuce, artichoke, chicory), medicinal (echinacea and chamomile), and ornamental value (chrysanthemum, dahlia, zinnia, gerbera, and others). The family is distributed all over the world, except in Antarctica [34]. The species of the Asteraceae family have pharmaceutical applications, as they possess antioxidant, anti-inflammatory, antimicrobial, diuretic, and wound-healing properties [35]. In addition, insecticidal [36] and fungicidal activity [37] has also been reported for their essential oils. The above activities are attributed to their phytochemical profile, which consists of terpenoids, lignans, saponins, polyphenolic compounds, phenolic acids, sterols, and polysaccharides [38]. Terpenoids and especially monoterpenes and sesquiterpenes are abundant [39]. Monoterpenes have been reported to act as AChE inhibitors in various insects [40], whereas sesquiterpene lactones have been characterized as constituents with great biological value [41].

### 2.3. Apiaceae

Apiaceae (or Umbelliferae) is a family of mostly aromatic annual, biennial, or perennial herbs and less often shrubs or trees. It consists of 442 genera and 3575 species and has a worldwide distribution mostly in the northern temperate regions and high altitudes in the tropics [42]. The family includes crops with nutritional, medicinal, and industrial use. They also can be used as beverages, spices, cosmetics, and fragrances [43]. The essential oils of many species have been exploited successfully for insecticidal activity [44], fungicidal [45], and herbicidal activity [46]. This activity is correlated with their chemical composition, which consists of more than 760 different constituents [47,48]. Monoterpenes, phthalides, terpenoids, phenylpropanoids (coumarins and phenylpropenes), and polyacetylenes are commonly found in Apiaceae plants [49].

### 2.4. Cistaceae

The Cistaceae family consists of 8 genera and 180 species (shrubs and herbs) distributed in temperate and subtropical regions of the northern hemisphere, especially the western Mediterranean region [50]. Five of the eight genera (*Cistus*, *Fumana*, *Halimium*, *Helianthemum*, and *Tuberaria*) are native to this region, whereas the remaining three (*Crocanthemum*, *Hudsonia*, and *Lechea*) are native to temperate regions in the Americas [51]. The phytochemical profile of the *Cistus* species and especially the high amounts of polyphenolic compounds (especially catechins) provide them with the ability to withstand extreme conditions [52]. The Cistaceae family also has a long history in medicine due to its pharmaceutical value (anti-inflammatory, antiulcerogenic, wound-healing, and antimicrobial properties). The main compounds of *Cistus* essential oils are monoterpenes (pinene, borneol, camphor, and carvacrol), sesquiterpenes (viridiflorol and zingiberene) and diterpenes (manoyl oxide and abietatriene) [53]. Species of the family have been examined successfully against the *Geotrichum candidum var. citri-aurantii* fungus in citrus [54].

### 2.5. Cupressaceae

The Cupressaceae family is a family of resinous, monoecious, and dioecious shrubs and trees (125 species) with a worldwide distribution [55]. The species present anti-inflammatory, anticancer, antimicrobial, insecticidal, and antifungal activity [24,56]. They mainly contain terpenes (monoterpenes and sesquiterpenes), alkaloids (piperidines), and polyphenols (phenolic acids, flavonoids, proanthocyanidins, lignans, acetophenones, and stilbenes). The species have an important role in drug development, and their phytochemicals can be used as a natural source for future drugs [57]. They also present significant repellent and insecticidal activity against various pests [58,59] and pathogens [60]. Juniper essential oils also showed promising results in weed control [61].

### 2.6. Brassicacae

The Brassicaceae family includes many economically important species that are cultivated for human food, animal feed, edible oil, and biofuel. A great number of weeds also belong to this family [62]. It consists of 3709 species and has a worldwide distribution, except in Antarctica [63]. The species contains a variety of secondary metabolites, and based on literature data, the the organosulphur compounds (glucosinolates), phenolic acids and flavonoids were found to be the most significant [64]. In particular, glucosinates provide benefits to human health by reducing risk for degenerative diseases but also in plant health by inducing resistance to insects and pathogens [65]. Morra et al. [66] and Konecka et al. [67] demonstrated the herbicidal and insecticidal activity of seed meal and oil, respectively, from *Sinapis alba* L.

**Table 1:** Overview of extraction methods and isolated compounds of Mediterranean plant species.

Family/Plant Species	Extraction Methods *	Major Isolated Compounds	References
Acanthaceae			
*Acanthus dioscoridis* L.	m	n.a. **	[44]
Amaranthaceae			
*Achyranthes aspera* L.	se	Flavonoids; saponins; tannins; steroids; cardiac glycosides; alkaloids; anthrequinones; terpenoids	[68]
Anacardiaceae	se		
*Pistacia atlantica* Desf.	h	EO leaves: terpinen-4-ol; (p)-cymene; α-pinene; spathulenol EO fruits: terpinen-4-ol; sabinene; α-pinene. EO bark: α-pinene; myrtenol; verbenol (rans-); β-pinene	[69]
*Pistacia atlantica* Desf.	h		[70]
*Pistacia khinjuk* Stocks.	h	Fruit oil: b-pinene; sabinene; leaf oil: spathulenol; b-pinene	[70]
*Pistacia lentiscus* L.	se	n.a.	[71]
Apiaceae			
*Anethum graveolens* L.	h	L-phellandrene; carvone; limonene	[72]
*Bifora radians* M. Bieb.	m	n.a.	[44]
*Carum carvi* L.	h	Carvone; D-limonene; α-myrcene; dihydrocarvone	[73]
*Carum carvi* L.	p	Limonene; carvone	[46]
*Carum carvi* L.	m, sub	(+) Carvone; d-limonene	[45]
*Coriandrum sativum* L.	m	n.a.	
*Crithmum maritimum* L.	h	Dill apiole; γ-terpinene; carvacrol methyl ether	[74]
*Crithmum maritimum* L.	h	Dillapiole; γ-terpinene (French EO), limonene; γ-terpinene (central Italy EO); thymol methyl ether; γ-terpinene (Sicilian EO)	[75]
*Cuminum cyminum* L.	h	α-Pinene; o-cymene; cuminaldehyde; ç-terpinene	[73]
*Cuminum cyminum* L.	p	Cuminic acid	[76]
*Daucus carota* L.	h	α-Pinene; β-pinene; borneol; myrcene	[77]
*Daucus lopadusanus* Tineo	m	n.a.	[78]
*Foeniculum vulgare* Mill.	h	Anethole	[79]
*Foeniculum vulgare* Mill.	h	α-Pinene; anethole; D-limonene; L-fenchone	[73]
*Foeniculum vulgare* Mill.	p	Trans-anethole; limonene; fenchone	[80]
*Helosciadium nodiflorum* (L.) W.D.J. Koch	h	Myristicin; (Z)-β-ocimene	[81]
*Heracleum sphondylium* L.	h	Octyl acetate; octyl butyrate; octyl hexanoate	[74]
*Pimpinella anisum* L.	h	Anethole; D-limonene; estragole; o-cymene	[73]
*Pimpinella anisum* L.	p	Transanethole	[80]
*Pimpinella anisum* L.	h	(E)-anethole; methyl chavicol	[74]
*Smyrnium olusatrum* L.	h	Curzerene; iso-furanodiene; furanoeremophil-1-one; germacrone; myrcene	[81]
Apocynaceae			
*Calotropis procera* (Aiton) W.T. Aiton	se	n.a.	[82]
*Nerium oleander* L.	m	n.a.	[83]
*Nerium oleander* L.	se	n.a.	[83]
Asclepiadaceae			
*Periploca angustifolia* Labill.	m	n.a.	[78]
Asphodelaceae		n.a.	
*Asphodelus ramosus* L. *subsp. ramosus*	m, ultra	n.a.	[58]
Asteraceae			[71]
*Achillea millefolium* L.	h	Chamazulene; 1,8-cineole	[36]
*Achillea millefolium* L.	m	n.a.	[44]
*Achillea millefolium* L.	m, sub	n.a.	[45]
*Achillea ptarmica* L.	m	n.a.	[84]
*Achillea millefolium* L.	m	n.a.	[84]
*Anthemis deserti* Boiss.	m	n.a.	[85]
*Arctium lappa* L.	m	n.a.	[84]
*Artemisia inculta* Delile	h	Camphor (19); 1,8-cineole (12); p-cymeneborneol	[28]
*Artemisia absinthium* L.	h	Sabinene (23.8%); β-myrcene (15.5%)	[36]
*Bidens tripartite* L.	m	n.a.	[84]
*Carduus acanthoides* L.	m	n.a.	[84]
*Carduus nutans* subsp. *leiophyllus* (Petrović) Stoj. & Stef.	m	n.a.	[84]
*Centaurea cyanus* L.	m	n.a.	[84]
*Centaurea jacea* L.	m	n.a.	[84]
*Centaurea scabiosa* L.	m	n.a.	[84]
*Cirsium arvense* (L.) Scop.	m	n.a.	[84]
*Cynara cardunculus* L. var. *altilis* DC.	m	Caffeoylquinic acids; apigenin; luteolins; lactone cynaropicrin	[86]
*Dittrichia viscosa* (L.) Greuter	m	α-Costic acid; inuloxin A	[87]
*Dittrichia viscosa* (L.) Greuter	n.a. **	α-Costic acid; inuloxin A; inuloxin C	[88]
*Echinops ritro* L. var. *tenuifolius*	m	n.a.	[84]
*Echinops spinosissimus* Turra	m	n.a.	[78]
*Gnaphalium uliginosum* L.	m	n.a.	[84]
*Glebionis coronaria* (L.) Spach	se	Camphor	[89]
*Leontodon hispidus* L.	m	n.a.	[84]
*Pentanema britannica* (L.) D. Gut. Larr., Santos-Vicente, Anderb., E. Rico & M.M. Mart. Ort.	m	n.a.	[84]
*Pulicaria crispa* (Forssk.) Oliv.	m	n.a.	[90]
*Santolina chamaecyparissus* L.	h	Artemisia ketone; β-phellandrene; vulgarone B; β-myrcene	[36]
*Santolina chamaecyparissus* L.	h	1,8-Cineole; 8-methylene-3-oxatricyclo [5.2.0.02,4] nonane	[91]
*Silybum marianum* (L.) Gaertn.	m	n.a.	[84]
*Sonchus arvensis* L.	m	n.a.	[84]
*Tanacetum vulgare* L.	m	n.a.	[92]
*Tanacetum vulgare* L.	h	α-Thujone; 1,8-cineole	[36]
*Taraxacum officinale* F.H. Wigg. subsp. *officinale*	m, sub	n.a.	[45]
*Tripleurospermum inodorum* (L.) Sch. Bip.	m	n.a.	[84]
*Solidago virgaurea* L.	h	Pentadecanol; germacrene D	[29]
Boraginaceae			
*Glandora prostrata* (Loisel.) D.C.Thomas	se	n.a.	[93]
*Onosma visianii* Clementi	se	Isobutylshikonin; isovalerylshikonin	[94]
Brassicaceae			
*Brassica rapa* L.	se	n.a.	[71]
*Diplotaxis erucoides* (L.) DC.	se	n.a.	[71]
*Diplotaxis virgata* (Cav.) DC.	se	n.a.	[71]
*Hirschfeldia incana* (L.) Lagr.-Foss.	se	n.a.	[71]
*Sinapis alba* L.	m	n.a.	[66]
Cannabaceae			
*Humulus lupulus* L.	m, sub	n.a.	[45]
*Humulus lupulus* L.	m	α-Humulene; myrcene; trans-caryophyllene	[95]
Caryophyllaceae			
*Saponaria officinalis* L.	m	n.a.	[96]
Chenopodiaceae			
*Atriplex halimus* L.	m	n.a.	[78]
*Chenopodium murale* (L.) S. Fuentes & al.	se	Flavonoids; saponins; tannins; steroids; cardiac glycosides; alkaloids; anthrequinones; terpenoids	[68]
Cistaceae			
*Cistus albidus* L.	se	n.a.	[71]
*Cistus albidus* L.	m	n.a.	[54]
*Cistus criticus* L.	m	n.a.	[54]
*Cistus crispus* L.	m	n.a.	[54]
*Cistus ladanifer* L.	se	n.a.	[71]
*Cistus ladanifer* L.	m	n.a.	[54]
*Cistus laurifolius* L.	se	n.a.	[71]
*Cistus laurifolius* L.	m	n.a.	[54]
*Cistus monspeliensis* L.	m	n.a.	[54]
*Cistus populifolius* L.	m	n.a.	[54]
*Cistus salviifolius* L.	m	n.a.	[54]
Convolvulaceae			
*Convolvulus arvensis* L.	se	Flavonoids; saponins; tannins; steroids; cardiac glycosides; alkaloids; anthrequinones; terpenoids	[68]
Cupressaceae			
*Juniperus communis* L.	h	α-Pinene; sabinene; β-myrcene; limonene; terpinen-4-ol; germacrene D; δ-cadinene	[59]
*Juniperus communis* L.	p	α-Pinene; myrcene	[60]
*Juniperus communis* L.	n.a.	α-Pinene; sabinene; limonene	[97]
*Juniperus communis* var. *saxatilis* Pall.	h	α-Pinene; sabinene; b-pinene; terpinen-4-ol; β-elemene	[59]
*Juniperus excelsa* M. Bieb.	h	α-Cedrol; α-limonene; α-pinene	[61]
*Juniperus oxycedrus* L.	h	α-Pinene; limonene; β-caryophyllene	[59]
*Juniperus phoenicea* L.	m, ultra	n.a.	[58]
*Juniperus sabina* L.	h	Sabinene	[61]
Dennstaedtiaceae			
*Pteridium αquilinum* (L.) Kuhn	m	Linolenic acid; phytol; palmitic acid; stearic acid; citronellol	[98]
Eqoisetaceae			
*Equisetum αrvense* L.	m, sub	n.a.	[45]
Fabaceae			
*Cassia senna* L.	m	n.a.	[85]
*Retama raetam* (Forssk.) Webb	m	Alpinumisoflavone; hydroxyalpinumisoflavone; laburnetin; licoflavone C; retamasin B; ephedroidin	[99]
*Sophora alopecuroides* L.	m	Alcaloids	[100]
*Ulex europaeus* L.	se	n.a.	[93]
Hypericaceae			
*Hypericum aegypticum* L.	m	n.a.	[78]
*Hypericum perforatum* L.	m, sub	n.a.	[45]
Juncaceae			
*Juncus compressus* Jacq.	p	Effusol; juncusol	[101]
Lamiaceae			
*Calamintha menthifolia* Host	m	Gallic acid; caffeic acid; 2-hidroxy-cinnamic acid; kaempferol; callistephin chloride; p-coumaric acid; idaenin chloride; (+)-catechin hydrate	[102]
*Hyssopus officinalis* L.	h	Cis-pinocamphone; b-phellandrene; b-pinene	[60]
*Hyssopus officinalis* L.	h	1,8-Cineole; b-pinene	[91]
*Lavandula intermedia* Emeric ex Loisel.	h	Linalyl acetate; linalool	[91]
*Lavandula angustifolia* Mill.	h	Linalyl acetate; linalool; geranyl acetate; terpineol	[28]
*Lavandula angustifolia* Mill.	h	Linalool; coumarin; α-terpineol; caryophyllene oxide; coumarin	[103]
*Lavandula angustifolia* Mill.	m, sub	n.a.	[45]
*Lavandula dentata* L.	h	Eucalyptol; fenchone; camphor	[104]
*Lavandula angustifolia* Mill.	n.a.	β-phellandrene; 1,8-cineole; terpinen-4-ol; caryophyllene	[97]
*Lavandula canariensis* Mill.	m	n.a.	[105]
*Melissa officinalis* L.	h	Geranial; neral; citronellal	[29]
*Mentha piperita* L.	m	n.a.	[106]
*Mentha piperita* L.	h	Menthone; menthol; limonene	[28]
*Mentha piperita* L.	h	Menthol; menthone	[46]
*Mentha piperita* L.	m, sub	n.a.	[45]
*Mentha piperita* L.	n.a.	Menthofuran; menthol	[97]
*Mentha spicata* L.	h	Carvone; 1,8-cineole; menthol	[28]
*Mentha spicata* L.	m	n.a.	[107]
*Mentha suaveolens* Ehrh.	h	Piperitenone oxide; bornel	[69]
*Mentha suaveolens* Ehrh.	h	Piperitenone oxide; piperitenone; limonene; D-germacrone; t-caryophyllene	[28]
*Mentha suaveolens* Ehrh.	m, ultra	n.a.	[58]
*Mentha x verticillata* L.	se	n.a.	[71]
*Mentha viridis* (L.) L.	m	n.a.	[85]
*Nepeta cataria* L.	h	n.a.	[108]
*Nepeta curviflora* Webb & Berthel.	h	2-Isopropyl-5-methyl-3-cyclohexen-1-one; (-)-spathulenol; cis-Z-α-bisabolene epoxide; widdrol; (E,Z)-5,7-dodecadiene; dihydronepetalactone; 4-propyl-cyclohexene	[109]
*Nepeta nuda* L. subsp. *pubescens*	h	Pinene; 1-ethyl-1H-pyrrole; 1-cycloethyl-1-(2-methylenecyclohexyl ethanol; 3-methyl-2-cyclohexen-1-one; 2,3-dimethyl-3-hexanol	[109]
*Origanum elongatum* (Bonnet) Emb. & Maire	h	Carvacrol; p-cymene; g-terpinene	[110]
*Origanum majorana* L.	h	n.a.	[108]
*Origanum syriacum* L. subsp. *syriacum*	h	Carvacrol	[25]
*Origanum virens* Hoffmanns. & Link	h	p-Cymene; carvacrol; linalool; a-terpinene; myrcene; b-caryophyllene	[28]
*Origanum vulgare* L.	h		[108]
*Origanum vulgare* L.	h	Terpinene; cis-p-menth-2-en-1-ol; terpinen-4-ol; thymol; α-terpinene	[111]
*Origanum vulgare* L.	se	n.a.	[71]
*Phlomis tuberosa* L.	m	n.a.	[44]
*Prasium majus* L.	M	n.a.	[78]
*Rosmarinus officinalis* L.	h	Verbenone, a-pinene	[112]
*Rosmarinus officinalis* L.	h	Camphor; 1,8-cineole; a-pinene; endoborneol; camphene; verbenone	[28]
*Rosmarinus officinalis* L.	h	Camphor; verbenone; eucalyptol (1,8-cineole)	[103]
*Rosmarinus officinalis* L.	n.a.	α-Pinene; linalool; piperitone	[97]
*Rosmarinus officinalis* L.	m	n.a.	[106]
*Rosmarinus officinalis* L.	m, sub	n.a.	[45]
*Salvia officinalis* L.	m	n.a.	[90]
*Salvia officinalis* L.	h	Thujone (trans); camphor; cineole,1,8	[110]
*Salvia officinalis* L.	h	Cis-thujone; camphor; viridiflorol; 1,8-cineole; trans-thujone; camphene; manool	[29]
*Salvia officinalis* L.	h	Camphor; thujone; isothujone	[103]
*Satureja hortensis* L.	h	Carvacrol; gamma-terpinene; paracymene	[72]
*Satureja hortensis* L.	h	Carvacrol; o-cymene; γ-terpinene; thymol	[113]
*Satureja hortensis* L.	m, sub	n.a.	[45]
*Satureja montana* L.	h	Carvacrol; p-cymene; borneol; thymoquinone; 1-octen-3-ol	[28]
*Satureja montana* L.	h	Carvacrol, followed by its precursor p-cymene	[114]
*Thymus leucotrichus* Halácsy	h	Thymol; p-cymene; g-terpinene; carvacrol	[28]
*Thymus leucotrichus* Halácsy	h	o-Cymene; α-pinene; ç-terpinene; camphene	[73]
*Thymus leucotrichus* Halácsy	h	p-Cymene; geraniol; thymol; carvacrol	[29]
*Thymus leucotrichus* Halácsy	p	Thymol; p-cymene; linalool; caryophyllene oxide	[26]
*Thymus leucotrichus* Halácsy	h	Thymol; p-cymene; γ-terpinene; caryophyllene oxide	[30]
*Thymus leucotrichus* Halácsy	h	Thymol; p-cymene; γ-terpinene	[60]
*Thymus leucotrichus* Halácsy	se	n.a.	[71]
*Thymus leucotrichus* Halácsy	m, sub	Thymol; p-cymene; carvacrol; γ-terpinene	[45]
*Thymus atticus* Čelak.	h	Carvacrol; o-cymene	[110]
*Thymus atticus* Čelak.	h	Thymol; p-cymene; g-terpinene; carvacrol	[28]
*Ziziphora clinopodioides* Lam.	h	Pulegone; piperitenone; isomenthone	[115]
Lauraceae			
*Laurus nobilis* L.	sfe	n.a.	[116]
*Laurus nobilis* L.	se	n.a.	[71]
Myrtaceae			
*Myrtus communis* L.	h	α-Pinene; 1,8-cineole	[79]
Oleaceae			
*Olea europaea* cv. Lechín de Sevilla	se	n.a.	[71]
*Olea europea* cv. Arbequina	se	n.a.	[71]
*Olea europea* cv. Cornicabra	se	n.a.	[71]
*Olea europea* cv. Empeltre	se	n.a.	[71]
*Olea europea* cv. Erantoio	se	n.a.	[71]
*Olea europea* cv. Picual	se	n.a.	[71]
Papaveraceae			
*Glaucium flavum* Crantz	m	n.a.	[78]
Poaceae			
*Echinochloa crus-galli* (L.) P. Beauv.	m	Loliolide; tricin	[117]
*Elytrigia repens* (L.) Nevski	m, sub	n.a.	[45]
Polygonaceae			
*Polygonum aviculare* L.	m, sub	n.a.	[45]
*Polygonum bistorta* (L.) Samp.	m, sub	n.a.	[45]
Pinaceae			
*Cedrus atlantica* (Endl.) Carrière	n.a.	α-Pinene; himachalane; β-himachalene	[97]
*Picea abies* (L.) H. Karst.	n.a.	Limonene; bornyl acetate; δ-cadinene; α-muurolol; δ-cadinol	[97]
*Pinus pinea* L.	se	n.a.	[71]
Plantaginaceae			
*Plantago albicans* L.	m	n.a.	[85]
Poaceae			
*Echinochloa crus-galli* (L.) P. Beauv.	m	Loliolide and tricin	[117]
Punicaceae			
*Punica granatum* L.	se	n.a.	[93]
Rosaceae			
*Prunus dulcis* (Mill.) D.A. Webb	n.a.	Fatty acids	[97]
Ranunculaceae			
*Nigella sativa* L.	m, sub	n.a.	[45]
Rutaceae			
*Ruta chalepensis* L.	m	n.a.	[105]
*Ruta chalepensis* L.		n.a.	[118]
*Ruta graveolens* L.	se	n.a.	[93]
Salicaceae			
*Populus nigra* L.	m	Alkanes; sterols; aliphatic and triterpenoic alcohols; acidic compounds	[119]
*Populus tremula* L.	m	n.a.	[120]
Solanaceae			
*Hyoscyamus niger* L.	m	Vanillic acid	[121]
*Solanum villosum* Mill.	m	n.a.	[85]
Urticaceae			
*Urtica dioica* L.	m	n.a.	[122]
*Urtica dioica* L.	m, sub	n.a.	[45]
*Urtica* sp.	se	n.a.	[71]
Verbenaceae			
*Lantana camara* L.	m	n.a.	[118]
Zygophyllaceae			
*Tribulus terrestris* L.	m	Flavonoids; saponins; tannins; steroids; cardiac glycosides; alkaloids; anthrequinones; terpenoids	[68]
*Zygophyllum eichwaldii* C.A. Mey.	m	n.a.	[85]

* Extraction methods: m: maceration, se: Soxhlet extraction, h: hydrodistillation, sub: subcritical fluid extraction, p: purchased or provided, ultra: ultrasound-assisted method, sfe: supercritical fluid extraction. ** n.a.: not available.

## 3. Extraction Methods and Determination of the Chemical Composition of Plant Extracts/Essential Oils

**Figure 1:**
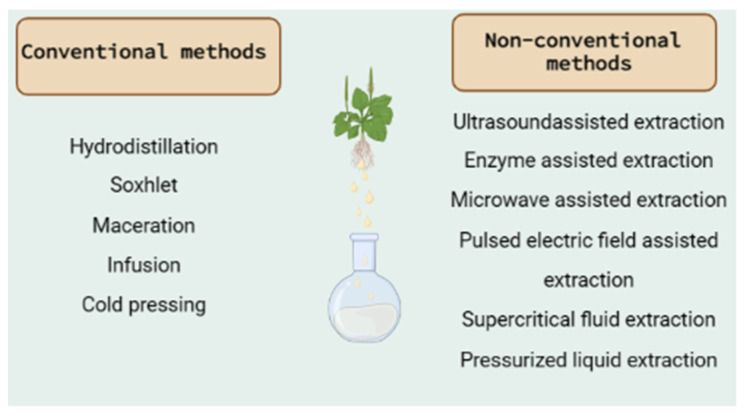
Conventional and non-conventional extraction methods [124,125].

The active compounds can be isolated from plant tissues with different extraction methods (Figure 1) using selective solvents. The extraction method is the first step to separating the active compounds from the raw material. The choice of extraction method is so crucial that it can affect further separation, as well as the chemical composition of the extracts [123]. 

In general, the features of the plant extracts and essential oils are dependent on the molecular weight and chemical types of the compounds extracted. The selection of the appropriate extraction method, as well as the appropriate conduction of the method, is important, as it can specify the quality and consequently the potential activity. For example, failure and error during the experimental procedure can lead to changes in chemical composition, discoloration, and off odor, reducing the overall quality of plant extracts and essential oils [13]. Moreover, raw material, the plant parts, solvent, temperature, pressure, and time are considered the most common factors affecting extraction processes [124].

Based on the literature data presented in Table 1, the most used methods are hydrodistillation, Soxhlet extraction or hot continuous extraction, and maceration. They belong to the conventional extraction methods that are mainly based on the extracting power of the different solvents and the application of heat and/or mixing [124]. The wide use of the conventional extraction techniques is based on the general advantages that they possess and include their simplicity, applicability at high temperatures, low investment cost, and selection of the appropriate solvent [125]. In contrast, their major limitations are the long extraction time, the requirement of high-purity solvents and the thermal decomposition of thermolabile compounds, and the poor extraction efficiency in some cases [124,125].

The basic characteristics of each extraction method, as well as the specific advantages and disadvantages, are reported below:

- Hydrodistillation: This is a traditional, simple method for the extraction of active compounds and especially essential oils from plants. Even though it can be used in fresh plant material, is is preferrable to use the method with dried plant material in order to preserve it from enzymatic degradation [126]. As some volatile components may be lost at high extraction temperatures, this method cannot be used for thermolabile compounds [127]. In this method, water and oil are exclusively separated through condensation to retain all the essential properties of the plant part used for the extraction [128]. It involves three main physicochemical processes: hydrodiffusion, hydrolysis, and heat decomposition [129]. Three types of hydrodistillation can be distinguished: (a) water distillation, (b) water and steam distillation, and (c) direct steam distillation [124]. Umpiérrez et al. [130] reported that the essential oils produced by different distillation methods did not differ in their chemical content in two Asteraceae plants. Hydrodistillation with the Clevenger-type apparatus has been used in most of the extractions, as can be seen in Table 1. It is a steam distillation technique with which the active compounds are extracted with the use of steam generated outside the tank in a steam generator or in a boiler. It can determine the percentage of volatile oils present in the oil-bearing material [131]. This method is preferred because (i) the released steam can easily be controlled and (ii) no thermal decomposition of oil constituents occurs because the temperature does not exceed 100 °C. On the other hand, it has been reported to require equipment that increases the cost of the method [128].
- Soxhlet extraction or hot continuous extraction: This is a continuous extraction method with high extraction efficiency that requires less time and solvent consumption than other methods (maceration or percolation) [132]. It is used for plant material that is partially soluble in the chosen solvent and for plant material with insoluble impurities [133]. There is also no need for filtration of the extract [126]. On the other hand, the device must not be shaken, and the long extraction time may lead to the degradation of thermolabile compounds [134].
- Maceration: This is a solid–liquid extraction and one of the most widely used techniques in the medicinal and aromatic plant industry. It is a separation technique to remove a solute from a solid mixture with the help of a solvent [126]. It is an appropriate method for thermolabile compounds [133]. The success of the method depends on the solvent, the plant part, and the starting material and extraction time. On the other hand, the large volume of solvents used and the long extraction time are the main disadvantages of the method [128].

**Figure 2:**
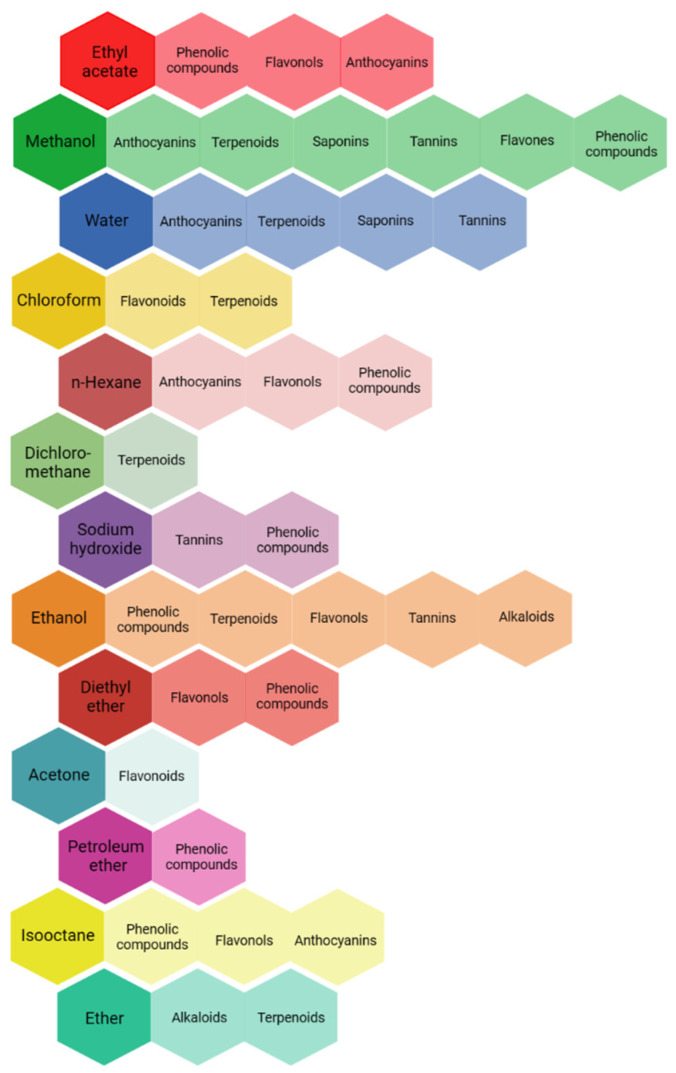
Frequently used solvents for the extraction of different active compounds [124,125].

The selection of the solvent is especially crucial amongst the factors previously reported for extraction. Solubility, selectivity, polarity, cost, and safety should be considered for the selection of the solvent [135]. Figure 2 shows different solvents used for the extraction of different active compounds from plant species. In general, methanol, ethanol, acetone, and water are preferred. Saaba et al. [136], analyzing the methanolic, ethanolic, acetonic, and aqueous extracts from different medicinal plants (such as *Juniperus phoenicea L.* and *Asphodelus microcarpus Salzm. & Viv.*), concluded that there were significant differences in the quantitative characterization of the different extracts depending on the solvent used. According to their results, the acetonic and methanolic extracts seemed to be most promising. The solvents have different polarities, and this affects the content of the active compounds, as well as their pesticidal activity. Water, methanol, and ethanol are used for the extraction of polar compounds (hydrophilic), whereas hexane and dichloromethane are used for the extraction of nonpolar compounds (lipophilic) [134,137].

Fractionation is also a widely used process that follows the extraction of raw material and aims to isolate specific compounds belonging mainly to the same chemical category. It is a continuous process that ends after the isolation of the compound of interest and demands several solvents, which are added based on their polarity (from less to more polar) [126,135]. Fractionation has been used for the isolation of alkaloids from *Sophora alopecuroides* L. extract [138], phenolic compounds from *Humulus lupulus* L. [95], and isoflavones and flavones from *Retama raetam* [99]. 

Qualitative and quantitative analysis of phytochemicals presented in extracts/essential oils can be performed using chromatographic and identification techniques [133]. Mass spectrometry (MS) is a powerful analytical tool that is used to identify unknown compounds and has been applied to a very wide range of areas, including biochemical sciences. Mass spectrometry provides abundant information for the structural elucidation of unknown compounds, especially when tandem mass spectrometry (MS/MS) is applied [139]. Most of the scientific works reported herein have used gas chromatography–mass spectroscopy (GC-MS) for phytochemical analysis of biopesticides [28,75,79]. It is a combined analytical technique that plays an essential role in the phytochemical analysis of plant extracts containing biologically active compounds [140]. Advantages of the technique include (i) the efficiency of gas chromatography separation, (ii) the good qualitative information and high sensitivity provided by mass spectrometry (MS), and (iii) the identification of unknown compounds by comparison with library spectra [141]. 

It is worth mentioning that high-performance liquid chromatography (HPLC) [90,94], liquid chromatography–mass spectroscopy (LC-MS) [87], and nuclear magnetic resonance (NMR) [87,121] have also been employed for the identification of secondary metabolites. The chromatographic and identification techniques have proven that the qualitative and quantitative variation of secondary metabolites in the same species depend on (i) genetic factors, (ii) environmental causes (light, temperature, soil water, soil fertility, and salinity), (iii) geographical origin, (iv) harvest stage, (v) part of the plant, (vi) processing modalities, and (vii) storage time [12,13,142]. 

## 4. Biological Activity of Plant Extracts and Essential Oils 

Literature data indicate that plant extracts have promising antimicrobial, insecticidal, and herbicidal activity. Key findings of several recent studies focusing on the antimicrobial, insecticidal, and herbicidal activity of Mediterranean plant extracts and essential oils are presented in detail in Table 2, Table 3 and Table 4. Their activity was also examined regarding plant bacteria, viruses, nematodes, and other pathogens (Table 5). Although numerous studies have evaluated the biological activity of plant extracts and essential oils, in most cases the observed activity was not correlated with specific components. The biological activity was attributed to the synergistic effects of the different compounds [28]. Nevertheless, there were cases where the biological activity was correlated with specific compounds. Indicatively, γ-terpinene and myristicin were found to possess insecticidal activity and were effective on *Culex quinquefasciatus* larvae [75].

It is also worth mentioning that, in some cases, the observed activity significantly varies for different targets and even the same targets between essential oils/extracts of the same plant. For example, Pavela et al. [75] investigated the essential oils of *Crithmum maritimum* L. of different geographical origins and observed a significant differentiation in their insecticidal activity due to their phytochemical compositions. Furthermore, the activity of the essential oils of different parts of the plant was also found to vary. In a recent study, Zerkani et al. [69] observed significant differences in antimicrobial activity from the essential oils derived from different parts of *Pistacia atlantica*.

In addition, the same active compound has been reported to possess varied biological activity. Oil containing thymol as a major component was found by Ben Jabeur et al. [30] to present antimicrobial properties. Essential oils with thymol have also been suggested as potential plant-based insecticidal agents [28]. Essential oils with carvacrol and piperitenone oxide as major compounds have also been suggested [28,113] and reported to possess insecticidal activity. Up to now, a variety of assays have been used to evaluate the biological activity, such as antimicrobial, insecticidal, herbicidal, etc., of plant extracts and essential oils, which are discussed in detail in the following sections.

### 4.1. Commonly Used Assays for Evaluating Antimicrobial Activity

Various methods are used to evaluate antimicrobial activity in vitro. Among them, the most common are the agar dilution and disc diffusion methods. Agar dilution, otherwise referred to as the poisoned food method, is the method of choice when estimating antifungal activity [143]. The method is based on preparing solid media and adding a desired concentration of the extract to it. A certain volume of the extract can be mixed before the autoclaved medium is poured on Petri dishes or spread on their surface once it has solidified [71,76,117]. Subsequently, a small agar plug (4–7 mm in diameter) from an active fungal culture is inverted, with the mycelial surface facing down, and inoculated at the center of the agar plate. The inhibition is estimated by measuring mycelial growth in optimal conditions and comparing it with a control sample [71]. One or multiple concentrations of the extract can be used during the assay. Different concentrations can be used to determine the potency of the antifungal effect by measuring certain indices, such as half maximal effective concentration (EC_50_) [76], the minimum inhibitory concentration (MIC), or the half inhibitory concentration (IC_50_) of the extract/essential oil [30,95]. Variations of the agar dilution method have been successfully employed to test the antifungal capacity of various extracts against plant pathogenic fungi, such as *Verticillium dahliae* in olives [71]; *Zymoseptoria tritici* in wheat [30,95]; *Sclerotinia sclerotiorum* [76], *Fusarium oxysporum*, *Alternaria solani*, and *Pythium ultimum* in tomato [106,117]; and *Botrytis cinerea* [116], *Penicillium allii* [111], *Stemphylium vesicarium* [99], and *Geotrichum candidum var. citri-aurantii* in decayed mandarin fruit [54]. Semerdjieva and colleagues used agar dilution to test the antifungal potential of essential oils against five fungal pathogens, including *Fusarium* sp. and *Rhizoctonia solani* strains isolated from stored potato, *Botrytis cinerea* from infected stored tomato, *Colletotrichum* sp. from anthracnose of bananas, and *Cylindrocarpon pauciseptatum* obtained from diseased grapevine [59]. Slight variations in the protocol involve inoculation of the agar containing the extract with a small volume from a liquid culture of the fungus [30,95] or with fungi-infected plant seeds [60] instead of an agar plug. Although the method is mostly used for fungal pathogens, Fu et al. [144] employed the agar dilution method to test the antibacterial potential of water extracts from aquatic weeds against 100 bacterial strains that were inoculated on agar plates by streaking. 

On the other hand, the disc diffusion method is mostly preferred when screening extracts for antibacterial activity in vitro. However, it can be used for testing antifungal activity as well [69]. This method is based on spreading an amount of bacterial or fungal suspension (or an agar plug from an active fungal culture) on solid media, placing small paper discs (5–6 mm in diameter) soaked with a microvolume of the extract (e.g., 3–5 μL), incubating the plates in ideal growth conditions, and measuring the inhibition zones [143]. Disc diffusion was used to assess both antifungal and antibacterial activity of three subcritical carbon dioxide plant extracts from *Carum carvi*, *Thymus vulgaris*, and *Nigella sativa* [45]. The extracts were successful at inhibiting eight fungal pathogens, including the *Fusarium*, *Alternaria*, *Colletotrichum*, *Rhizoctonia*, and *Phoma* strains, as well as two bacterial phytopathogens belonging to the genera *Pectobacterium* and *Streptomyces* [45]. The study also employed another in vitro assay to test antimicrobial activity, the agar well diffusion method, which shares many similarities to the disc diffusion method. In its most common form, a volume (e.g., 50–250 μL) of the extract is applied in a central well (5–8 mm in diameter) on the agar plate, which is previously inoculated with the pathogen. Twenty-two water and water–glycol extracts were tested by this method for antimicrobial effect against the 10 previously mentioned plant pathogens [45]. The disc diffusion method was used to assess the antifungal capacity of essential oils from *Lavandula dentata* against strains of *Cercospora kikuchii*, *Cercospora sojina*, and *Septoria glycines* [104]; of pyroligneous acids identified in the bark of hybrid aspen trees against *Fusarium culmorum* [120]; and of extracts from seven plant species collected from the island of Lampedusa, in Italy, against *Penicillium italicum*, *Aspergillus carbonarius*, and *Drechslera gigantea* [78]. It was also used to test the antibacterial effect of nano-suspensions of *Chrysanthemum coronarium* and *Azadirachta indica* against *Escherichia coli* and *Staphylococcus aureus* strains [89] and of barnyard grass extracts against a tomato bacterial pathogen, *Pectobacterium carotovorum* [117]. Other applications of the method include screening against human pathogens. For instance, essential oils extracted from the aerial parts of *Origanum elongatum* were tested against nine pathogenic bacteria isolated from hospital patients [110], whereas essential oils from *Pistacia atlantica* were assayed against 12 human pathogens, 9 bacterial strains and 3 fungal strains [69]. 

In vitro methods comprise the most common assays for antimicrobial screening since they are simple in terms of design and execution and provide useful and comprehensive results. On the other hand, in vivo and in situ assays are more challenging to set up and are thus less frequently used but generally provide more reliable data. Such an in situ antimicrobial assay was carried out by Steglińska and colleagues on potatoes [45]. In brief, water and subcritical carbon dioxide extracts (SCDE) from four plant species exhibited antifungal and antibacterial effects when they were applied on potatoes. The in situ assay included immersion of potatoes in the plant extracts, application of 20 μL of bacterial or fungal suspension in three cuts (5 mm in diameter and 5 mm deep), and measuring the infestation rate after 2 weeks of incubation [45]. A similar test was conducted by Karim and colleagues, who created 2 mm-deep and 3 mm-wide wounds on mandarin fruit with sterile needles [54]. The cuts were inoculated with 30 mL of *Cistus* aqueous extract and 20 mL of a *Geotrichum candidum var. citri-aurantii* suspension. The incidence and severity of the fungal disease on the treated mandarin fruit was evaluated daily for 10 days [54]. Regarding antiviral activity, Hu et al. employed the half-leaf method to test the effect of nine compounds from the seeds of *Hyoscyamus niger* against a phytopathogenic virus, tobacco mosaic virus (TMV) [121]. The method is often used to test inactivation, protective, and curative effects of extracts against the selected pathogen and is based on smearing half of the surface of the leaf with the extract while leaving the other side with a control treatment. Depending on the type of effect that is being tested, the viral suspension is either mixed with the compounds and applied on the same side of the leaf or inoculated on the whole surface of the leaf [145]. 

**Table 3:** Recent studies on insecticidal activity of Mediterranean plant extracts/essential oils.

Insects Tested	Family	Plant	Parts Used for Extraction	References
*Acrobasis advenella*	Lamiaceae	*Satureja hortensis* L.	Aerial parts	[113]
*Acromyrmex octospinosus*	Apocynaceae	*Nerium oleander* L.	Leaves	[83]
*Aedes aegypti* L.	Apiaceae	*Daucus carota* L.	Umbels	[77]
*Amblyseius swirskii*	Lamiaceae	*Satureja hortensis* L.	Aerial parts	[72]
*Myzus persicae*	Asteraceae	*Artemisia absinthium* L.	Aerial parts	[36]
		*Santolina chamaecyparissus* L.	Aerial parts	
		*Tanacetum vulgare* L.	Aerial parts	
	Compositeae	*Achillea millefolium* L.	Aerial parts	
	Fabaceae	*Sophora alopecuroides* L.	Aerial parts	[100]
	Lamiaceae	*Origanum syriacum* L. subsp. *syriacum*	Leaves	[25]
	Lamiaceae	*Satureja montana* L.	Leaves and flowers	[114]
Experimental model of aphids’ nervous system	Lamiaceae	*Lavandula angustifolia* Mill.	Aerial parts	[103]
		*Satureja montana* L.	Aerial parts	
		*Salvia officinalis* L.	Aerial parts	
*Aphis craccivora*	Resedaceae	*Ochradenus baccatus* Delile	Leaves	[90]
	Asteraceae	*Pulicaria crispa* (Forssk.) Oliv. (Forssk.) Oliv.	Leaves	
	Lamiaceae	*Salvia officinalis* L.	Leaves	
*Apis mellifera*	Asteraceae	*Artemisia absinthium* L.	Aerial parts	[130]
*Aphis citricola*	Fabaceae	*Sophora alopecuroides* L.	Aerial parts	[100]
*Macrosiphum rosirvorum*	Fabaceae	*Sophora alopecuroides* L.	Aerial parts	[100]
*Sitobion avenae*	Cupressaceae	*Juniperus communis* L.	n.a. *	[59]
*Brevicoryne brassicae*		*Juniperus oxycedrus* L.	n.a.	
*Brassicogethes aeneus*		*Juniperus communis* var. *satilis* Pall.	n.a.	
*Callosobruchus maculatus*	Anacardiaceae	*Pistacia atlantica* Desf.	Fruits, leaves and gum	[70]
		*Pistacia khinjuk* Stocks	Fruits and leaves	
*Ceratitis capitata*	Labiatae	*Origanum elongatum* (Bonnet) Emb. & Maire	Aerial parts	[110]
	Anacardiaceae	*Pistacia atlantica* Desf.	n.a.	[69]
	Lamiaceae	*Mentha suaveolens* Ehrh.	n.a.	[146]
		*Salvia officinalis* L.	n.a.	
		*Thymus atticus* Čelak.	n.a.	
*Chaitophorus populialbae*	Dennstaedtiaceae	*Pteridium aquilinum* (L.) Kuhn	Leaves	[98]
*Chrysoperla carnea*	Lamiaceae	*Salvia officinalis* L.	Leaves	[90]
	Resedaceae	*Ochradenus baccatus* Delile	Leaves	
	Asteraceae	*Pulicaria crispa* (Forssk.) Oliv.	Leaves	
*Culex pipiens* L.	Apiaceae	*Daucus carota* L.	n.a.	[77]
*Culex quinquefasciatus*	Apiaceae	*Smyrnium olusatrum* L.	Umbels	[81]
		*Helosciadium nodiflorum* (L.) W.D.J. Koch	Aerial parts	
	Chenopodiaceae	*Chenopodium murale* (L.) S. Fuentes et al.	Whole plant	[68]
	Amaranthaceae	*Achyranthes aspera* L.	Whole plant	
	Zygophyllaceae	*Tribulus terrestris* L.	Whole plant	
	Convolvulaceae	*Convolvulus arvensis* L.	Whole plant	
	Apiaceae	*Crithmum maritimum* L.	Aerial parts, leaves, flowers, and seeds	[75]
	Lamiaceae	*Ziziphora clinopodioides* Lam.	Aerial parts	[115]
*Culex restuans* Theobald	Apiaceae	*Daucus carota* L.	Umbels	[77]
*Cydia pomonella* L.	Cannabaceae	*Humulus lupulus* L.	n.a.	[67]
*Dendrolimus pini* L.	Brassicaceae	*Sinapis alba* L.	n.a.	[67]
*Diaphorina citri*	Asteraceae	*Artemisia absinthium* L.	Leaves and flowers	[147]
*Epicauta atomaria*	Lamiaceae	*Lavandula dentata* L.	Leaves and green stems	[104]
*Harmonia axyridis*	Lamiaceae	*Origanum syriacum* L. subsp. *syriacum*	Leaves	[25]
*Leptinotarsa decemlineata*	Lamiaceae	*Phlomis tuberosa* L.	Stems, leaves, and flowers	[44]
	Apiaceae	*Bifora radians* M. Bieb.	Leaves and stems	
	Apiaceae	*Heracleum platytaenium* Boiss.	Leaves and stems	
	Acanthaceae	*Acanthus dioscoridis* L.	Stems, leaves, and flowers	
	Cannabaceae	*Humulus lupulus* L.	Cone	
	Asteraceae	*Achillea millefolium* L.	Stems, leaves, and flowers	
	Lamiaceae	*Satureja montana* L.	Leaves and flowers	[114]
	Asteraceae	*Santolina chamaecyparissus* L.	Aerial parts	[91]
	Lamiaceae	*Hyssopus officinalis* L.	Aerial parts	
	Lamiaceae	*Lavandula intermedia* Emeric ex Loisel.	Aerial parts	
*Macrosiphum euphorbiae*	Apiaceae	*Foeniculum vulgare* Mill. Mill.	n.a.	[80]
	Apiaceae	*Pimpinella anisum* L.	n.a.	
*Musca domestica*	Lamiaceae	*Origanum syriacum* L. subsp. *syriacum*	Leaves	[25]
*Phthorimaea operculella*	Plantaginaceae	*Plantago albicans* L.	n.a.	[85]
	Solanaceae	*Solanum villosum* Mill.	n.a.	
	Zygophyllaceae	*Zygophyllum eichwaldii* C.A. Mey.	n.a.	
*Rhopalosiphum maidis*	Apiaceae	*Foeniculum vulgare* Mill.	n.a.	[79]
	Myrtaceae	*Myrtus communis* L.	n.a.	
*Rhopalosiphum padi*	Cupressaceae	*Juniperus communis* L.	n.a.	[59]
	Cupressaceae	*Juniperus oxycedrus* L.		
	Cupressaceae	*Juniperus pygmaea* M.-Bieb.		
	Lamiaceae	*Hyssopus officinalis* L.	Aerial parts	[91]
	Lamiaceae	*Lavandula intermedia* Emeric ex Loisel.	Aerial parts	
	Asteraceae	*Santolina chamaecyparissus* L.	n.a.	
*Rhyzopertha dominica*	Asteraceae	*Glebionis coronaria* (L.) Spach	n.a.	[89]
*Sitophilus oryzae*	Lamiaceae	*Mentha longifolia* (L.) Huds.	n.a.	[148]
*Sitophilus zeamais*	Lamiaceae	*Lavandula dentata* L.	Leaves and green stems	[104]
*Spodoptera exigua*	Brassicaceae	*Sinapis alba* L.	n.a.	[67]
*Spodoptera frugiperda*	Fabaceae	*Ulex europaeus* L.	Leaves and flowers	[93]
	Punicaceae	*Punica granatum* L.	Fruit peel	
	Rutaceae	*Ruta graveolens* L.	Leaves	
	Boraginaceae	*Glandora prostrata* (Loisel.) D.C. Thomas	Leaves and flowers	
	Labiatae	*Origanum majorana* L.	Leaves and stems	[108]
	Lamiaceae	*Nepeta cataria* L.	Leaves and stems	
		*Origanum vulgare* L.	Leaves and stems	
	Lythraceae	*Punica granatum* L.	Fruit peel	
*Spodoptera littoralis*	Labiatae	*Origanum virens* Hoffmanns. & Link	Aerial parts	[28]
	Lamiaceae	*Lavandula angustifolia* Mill.	Aerial parts	
	Lamiaceae	*Satureja montana* L.	Aerial parts	
	Lamiaceae	*Thymus leucotrichus* Halácsy	Aerial parts	
	Lamiaceae	*Thymus atticus* Čelak.	Aerial parts	
	Lamiaceae	*Mentha piperita* L.	Aerial parts	
	Lamiaceae	*Satureja montana* L.	Aerial parts	
	Lamiaceae	*Mentha spicata* L.	Aerial parts	
	Lamiaceae	*Mentha suaveolens* Ehrh.	Aerial parts	
	Asteraceae	*Artemisia inculta* Delile	Aerial parts	
	Lamiaceae	*Origanum syriacum* L. subsp. *syriacum*	Aerial parts	[25]
	Lamiaceae	*Satureja montana* L.	Aerial parts	
	Lamiaceae	*Hyssopus officinalis* L.	Aerial parts	[91]
	Lamiaceae	*Lavandula intermedia* Emeric ex Loisel.	Aerial parts	
	Asteraceae	*Santolina chamaecyparissus* L.	Aerial parts	
*Tetranychus cinnabarinus*	Asteraceae	*Artemisia capillaris* Thunb.	n.a.	[149]
*Tetranychus turkestani*	Lamiaceae	*Mentha longifolia* (L.) Huds. L.	n.a.	[150]
	Lamiaceae	*Rosmarinus officinalis* L.	n.a.	
*Tetranychus urticae*	Lamiaceae	*Satureja hortensis* L.	Aerial parts	[72]
	Apiaceae	*Anethum graveolens* L.	Aerial parts	
	Boraginaceae	*Onosma visianii* Clementi	Roots	[94]
	Caryophyllaceae	*Saponaria officinalis* L.	n.a.	[96]
*Thrips tabaci*	Lamiaceae	*Satureja montana* L.	Leaves and stems	[112]
*Trialeurodes vaporariorum*	Asteraceae	*Artemisia absinthium* L.	Aerial parts	[130]
*Tribolium castaneum*	Cupressaceae	*Juniperus phoenicea* L.	Leaves	[58]
		*Cupressus sempervirens* L.	Leaves	
	Asphodelaceae	*Asphodelus microcarpus* Salzm. & Viv.	Leaves	
	Lamiaceae	*Mentha rotundifolia* (L.) Huds	Leaves	
	Lamiaceae	*Lavandula dentata* L.	Leaves and green stems	[104]
	Asteraceae	*Glebionis coronaria* (L.) Spach	Leaves and flowers	[89]
	Lamiaceae	*Mentha spicata* L.	Plant samples	[107]
*Tribolium confusum*	Lamiaceae	*Lavandula angustifolia* Mill.	n.a.	[97]
	Lamiaceae	*Mentha piperita* L.	n.a.	
	Lamiaceae	*Satureja montana* L.	n.a.	
	Pinaceae	*Picea abies* (L.) H. Karst.	n.a.	
	Rosaceae	*Prunus dulcis* (Mill.) D.A. Webb	n.a.	
*Trichoplusia ni*	Lamiaceae	*Thymus leucotrichus* Halácsy	n.a.	[26]
*Trogoderma granarium*	Rutaceae	*Ruta chalepensis* L.	Aerial parts	[118]
	Verbenaceae	*Lantana camara* L.	Aerial parts	
	Apocynaceae	*Calotropis procera* (Aiton) W.T. Aiton	Leaves	[82]
*Tuta absoluta*	Asteraceae	*Tanacetum vulgare* L.	Flowers, leaves, and buds	[92]
	Lamiaceae	*Mentha suaveolens* Ehrh.	n.a.	[146]
	Lamiaceae	*Salvia officinalis* L.	n.a.	[110]
	Lamiaceae	*Thymus atticus* Čelak.	n.a.	
	Anacardiaceae	*Pistacia atlantica* Desf.	Leaves, fruit, and barks	[69]
	Asteraceae	*Tanacetum vulgare* L.	Flowers, leaves, and buds	[92]

* n.a.: not available.

### 4.2. Bioassays for Determining Pesticidal or Repellent Activity

Plant extracts can be submitted to a variety of assays to evaluate their insecticidal, acaricidal, nematocidal, or repellent potential, as well as their effect on oviposition. Standardized techniques include topical application, residual or surface contact, immersion in the extract or in a solution containing the extract, feeding bioassays, and fumigation [80,151]. Usually, the selected assay takes into consideration the unique biology of each pest or its developmental stage, since the egg and larval stages have different morphological and biological characteristics than the adult stage. 

Among the previously mentioned techniques, topical application can be used for bioassays in most developmental stages. The technique is based on applying microvolumes of the extract directly on the body of the insect with a micropipette or a microsyringe [151]. It was used successfully for larvae of the lepidopteran *Spodoptera littoralis*. Different concentrations of *Origanum syriacum* subsp. s*yriacum* extract were mixed with 1 μL of acetone, and each solution was applied on the dorsal region of 80 larvae per dose [25]. Insecticidal bioassays using topical application of extracts with a microsyringe were similarly performed on the dorsal region of *Spodoptera frugiperda* larvae [108]. Topical application tests can also be performed on adult individuals. However, in this case, since adults of certain insects display high motility or flying ability, as a first step before the topical application of the extract, the insects are anaesthetized with CO_2_ or on ice [25,88,152]. For instance, female *Musca domestica* flies were first anaesthetized and then treated with different doses of *Origanum syriacum* subsp. s*yriacum* extracts by applying a microvolume of the extract on the pronotum of the flies and measuring the effect after 24 h [25]. Topical application methods have been used to assay multiple insect species, such as *Pectinophora gossypiella*, *Thaumatotibia leucotreta*, *Helicoverpa armigera*, *Myzus persicae*, *Aphis craccivora*, *Aphis citricola*, *Aedes aegypti*, *Diaphorina citri*, *Tribolium castaneum*, *Trichoplusia ni*, and *Brassicogethes aeneus*. [26,58,73,88,100,147,152]. In the case of *Trichoplusia ni* larvae, an injection assay was also performed, with one microliter of test solution injected into the ventral hemocoel [26].

On the other hand, during residual contact techniques, individuals or groups of target organisms are exposed to residues of the bioactive compounds. The compounds are usually added uniformly on natural (e.g., leaves, fruit, inflorescences) or artificial (e.g., filter discs) surfaces, and the specimens are placed on them [151]. Such a residual contact assay was applied by Alkan and Gökçe [44] on egg masses of the Colorado potato beetle *Leptinotarsa decemlineata*. The eggs that were oviposited on potato leaflets were sprayed with 20 μL of six plant extracts to examine their ovicidal effect. The leaflets were then placed in petri dishes and egg mortality was recorded for 7 days [44]. Residual spraying was also used to apply plant essential oils on adult aphids (*Myzus persicae*) [36]. Other surface contact techniques that did not employ spraying were used to determine the acaricidal efficacy of different concentrations of an extract from *Onosma visianii* roots [94]. The mite that was subjected to the treatment belonged to the species *Tetranychus urticae*. A pipette was used to apply 20 μL of the various dilutions on one side of bean leaf discs (sized 2 cm^2^), which were then placed on agar-containing plates. Various developmental stages of the mites were assayed. Adult females, nymphs, or eggs were transferred to the discs and incubated at fixed temperature and light conditions for 24 h or for up to five days after the treatment. Thus, this assay, with minor modifications for each case, was used to assess adult mortality, the number of oviposited eggs for live females, and the hatchability of eggs [94]. A similar study was carried out for *Saponaria officinalis*-synthesized silver nanocrystals against *Tetranychus urticae* [96]. Surface toxicity was also used to assess the larvicidal activity of *Tagetes minuta* essential oils to *Lucilia cuprina* flies. The applied protocol was based on transferring third-instar larvae of the fly in glass vials with filter papers impregnated with different dilutions of the essential oils [153]. Various residual or surface contact bioassays, with certain modifications in their protocols, were used to test the bioactivity of a variety of plant extracts and essential oils against eggs, larvae, and adult specimens of insects and mites [70,72,74,82,87,89,90,92,98,102,113,130,147,148,150,154,155,156,157,158,159]. For instance, Erdogan and Mustafa dipped tomato leaf discs into the test solutions instead of pipetting a volume onto their surface and then placed *Tuta absoluta* larvae on them [92]. Surface contact bioassays can be performed not only on a laboratory scale but also on a larger scale. For instance, extracts from leaves of *Agave americana* were used against the hemipteran *Brevicoryne brassicae* in field experiments performed at a cabbage farm. The application of the extracts was carried out by spraying parts of the leaves and the center of the adult plant [156]. 

Repellency, rather than acute toxicity or pest mortality, may also be assessed with modified surface contact methods. Typical repellency assays use filter papers that are treated with the extract on one half and the respective solvent on the other half and are subsequently placed in Petri dishes with the test samples [107]. Such repellency bioassays were carried out for larvae of the khapra beetle, *Trogoderma granarium* [82], and adults of *Tribolium castaneum* [107]. Ilyas and colleagues, on the other hand, treated guava fruits by immersing them in plant extract solutions. The treated fruits were subsequently offered to adult *Bactrocera zonata* flies that were kept in cages, and the number of individuals that settled on the fruits were recorded for 5 h per day for two days [154]. Mangang and colleagues also used a more sophisticated system, termed an “insect management unit,” to study the repellent properties of packaging material [107]. Pourya et al. also used an arena to perform repellency bioassays on adult *Callosobruchus maculatus* beetles [70]. The arena consisted of three plastic chambers that were connected by small tubes. The beetles were placed in the central chamber, the control cowpeas treated only with solvents were placed in the first test chamber, and the cowpeas that were treated with different concentrations of *Pistacia* essential oils were placed in the second test chamber [70]. 

Immersion techniques are especially suitable for developmental stages that take place within an aquatic environment, such as eggs or larvae of certain species. Therefore, immersion assays were performed on larvae of *Culex quinquefasciatus* mosquitoes [68,75,81,115]. The larvae were placed in 250 mL of solution containing 249 mL of distilled water and 1 mL of essential oils or a mixture (six different dosages were tested for each compound), and their mortality was recorded after 24 h of exposure to the treatment [81]. Similar approaches were used in other studies featuring larvae of other mosquito species, such as *Culex pipiens*, *Culex restuans*, *Aedes aegypti*, *Aedes albopictus,* and *Anopheles gambiae* [77,82,88,160,161]. Musso and colleagues used immersion techniques to study the larvae of the nematode *Panagrolaimus rigidus* [109]. Briefly, they placed 100 μL of suspension containing approximately 100 larvae in each well of a 96-well microplate. Then, they added 100 μL of essential oil solutions isolated from *Nepeta* plant species and incubated the microplates at 20 °C. Nematocidal activity was estimated by counting mobile and immobile roundworms using an optical microscope [109]. Immersion bioassays can be also performed to test the activity of extracts on insect eggs [162]. Eggs of the lepidopteran *Conopomorpha sinensis* were submerged in two different concentrations of various plant extracts for 10 s, and their hatching rate was measured for two days [162]. The use of solid formulations against the potato tuber moth *Phthorimaea operculella* can be considered a modified case of immersion methods [85]. The process was based on crude extracts that were mixed with talcum powder (magnesium silicate) as an inert carrier substrate. Moths were completely covered with the powdered extract, which was firmly attached to their cuticle. Mortality and other biological parameters of the moths were recorded after the application of the powder [85]. Immersion-based assays were carried out to study nematocidal activity against other species of nematodes, such as *Meloidogyne incognita* [122] and *Meloidogyne javanica* [91,114], as well as acaricidal activity against *Tetranychus cinnabarinus* mites using the slip-dip method [149]. 

Feeding bioassays were performed against adult aphids of the species *Myzus persicae*. In this case, different concentrations of *Origanum syriacum* subsp. s*yriacum* extracts were applied on cabbage, and 4 groups of 50 individuals were left to feed on it. Mortality was recorded 48 h after the application of the treatment [25]. Similar feeding assays were conducted for the leaf-cutting ants *Acromyrmex octospinosus* using extracts from *Mammea americana* seeds and *Nerium oleander* and *Nicotiana tabacum* leaves [83]. The insecticidal activity of *Brassica alba* mustard oil against the lepidopteran species *Cydia pomonella*, *Dendrolimus pini*, and *Spodoptera exigua* [67], as well as of *Eucalyptus* essential oils on *Sitophilus oryzae* and *Sitophilus granarius* [163], was also assessed by feeding bioassays. Feeding inhibition caused by *Satureja montana* essential oils was measured for *Spodoptera littoralis* larvae and *Myzus persicae* and *Leptinotarsa decemlineata* adults. The antifeedant activity was calculated by measuring the consumption of treated leaf discs and comparing it with the controls [114]. Different concentrations of extracts can be mixed and tested not only with a natural host but also with artificial larval diets. Such was the case of *Spodoptera frugiperda* (fall armyworm) larvae that were submitted to various concentrations of extracts from the aerial parts of *Senna crotalarioides* plants [164]. Similar feeding inhibition assays were conducted with other extracts isolated from various plant species, such as *Hyssopus officinalis*, *Lavandula intermedia*, and *Santolina chamaecyparissus* [91]; 14 plant species belonging to the families Asteraceae and Lamiaceae [28]; and with trans-anethole compounds from various Apiaceae species [157]. 

Fumigant bioassays can be conducted for volatile organic compounds. For instance, volatile essential oils isolated from bitter fennel (*Foeniculum vulgare*) and green anise (*Pimpinella anisum*) were tested for insecticidal activity against *Macrosiphum euphorbiae* aphids, which infest tomatoes [80]. The tested essential oils were applied on filter papers, and the experiment was conducted on a small scale (only on tomato leaflets) and on a large scale both with whole plants and at the greenhouse level [80]. A different setup was used to test the insecticidal activity of lemongrass and rosemary essential oils against onion thrips, *Thrips tabaci*. Small *Allium schoenoprasum* seedlings with approximately 20 leaves were inserted separately into 50 mL test tubes. One milliliter glass tubes containing the essential oils were placed in each test tube along with 10 adult thrips for three days, and the mortality rate was calculated [112]. Other cases of fumigant bioassays with plant extracts and volatile essential oils have also been documented [70,74,150,163].

It is crucial for novel biopesticides to show high specificity and activity only against their intended target pests. For that reason, similar bioassays can be executed to assess the safety of the compounds against non-target organisms, such as the ladybug *Harmonia axyridis*, *Eisenia fetida* earthworms, the green lacewing *Chrysoperla carnea*, honeybees, or *Trichogramma pretiosum* hymenoptera [25,90,108,130]. Non-target organisms may also include predatory mites, such as the species *Amblyseius swirskii*, which is widely used as a natural enemy for biological control of small pest species, including mites, thrips, and whiteflies [72]. Similarly, Pino-Otín and colleagues assessed the ecotoxicological impact of a biopesticide from *Artemisia absinthium* on the soil microbial communities of the earthworm *Eisenia fetida* and the plant *Allium cepa*. The changes in microbial communities were assessed with metagenomic amplicon sequencing of 16S rRNA, and toxicity tests on the onion plant were conducted on young bulbs. For the nematocidal assay, they estimated mortality by placing 10 adult earthworms on 500 gr of soil in 1 L plastic containers treated with different concentrations of the aqueous extract [165]. 

**Table 4:** Recent studies on herbicidal activity of Mediterranean plant extracts/essential oils.

**Weeds Tested**	**Family**	**Plant**	**Parts Used for Extraction**	**References**
*Abutilon theophrasti* Medik.	Compositeae	*Solidago virgaurea* L.	Leaves and flowers	[29]
	Lamiaceae	*Melissa officinalis* L.	Leaves	
		*Salvia officinalis* L.	Leaves and flowers	
		*Thymus leucotrichus* Halácsy	Arial parts	
*Amaranthus powellii* S. Watson	Brassicaceae	*Sinapis alba* L.	Seeds	[66]
*Amaranthus retroflexus* L.	Asteraceae	*Cynara cardunculus* L.	Leaves	[86]
*Amaranthus spinosus* L.	Poaceae	*Echinochloa crus-galli* (L.) P. Beauv.	Leaves	[117]
*Anagallis arvensis* L.	Asteraceae	*Cynara cardunculus* L.	Leaves	[86]
*Brassica rapa* L.	Salicaceae	*Populus tremula* L.	Bark mass, including both inner and outer layers	[120]
*Capsicum annuum* L.	Lamiaceae	*Calamintha menthifolia* Host	n.a. *	[102]
*Cyperus iria* L.	Poaceae	*Echinochloa crus-galli* (L.) P. Beauv.	Leaves	[117]
*Echinochloa crus-galli* (L.) P. Beauv.	Apiaceae	*Carum carvi* L.	Seeds	[46]
	Apiaceae	*Mentha piperita* L.	n.a.	
*Lolium perenne* L.	Asteraceae	*Santolina chamaecyparissus* L.	Aerial parts	[91]
	Lamiaceae	*Hyssopus officinalis* L.	Aerial parts	
		*Lavandula intermedia* Emeric ex Loisel.	Aerial parts	
*Melilotus officinalis* L.	Cupressaceae	*Juniperus excelsa* M. Bieb.	Leaves	[61]
	Cupressaceae	*Juniperus sabina*	Leaves	[61]
*Myosotis arvensis* (L.) Hill	Cupressaceae	*Juniperus excelsa* M. Bieb.	Leaves	[61]
		*Juniperus sabina* L.	Leaves	
*Orobanche cumana* Wallr.	Fabaceae	*Retama raetam* (Forssk.) Webb	Aerial parts	[99]
*Portulaca oleracea* L.	Asteraceae	*Cynara cardunculus* L.	Leaves	[86]
*Setaria viridis* (L.) P. Beauv.	Brassicaceae	*Sinapis alba* L.	Seeds	[66]
*Solanum nigrum* L.	Lamiaceae	*Clinopodium menthifolium* (Host)		[102]
*Stellaria media* (L.) Vill.	Asteraceae	*Cynara cardunculus* L.	Leaves	[86]
*Trigonella besseriana* Ser.	Cupressaceae	*Juniperus excelsa* M. Bieb.	Leaves	[61]
		*Juniperus sabina* L.	Leaves	
**Plants Tested for Phytotoxicity**	**Family**	**Plant**	**Parts Used for Extraction**	**References**
*Solanum lycopersicum* L. (Mirella and Cetia seeds)	Lamiaceae	*Prasium majus* L.	n.a.	[78]
	Papaveraceae	*Glaucium flavum* Crantz		
	Apiaceae	*Daucus lopadusanus* Tineo		
	Asclepiadaceae	*Periploca angustifolia* Labill.		
	Asteraceae	*Echinops spinosissimus* Turra		
	Chenopodiaceae	*Atriplex halimus* L.		
	Clusiaceae	*Hypericum aegypticum* L.		
	Asteraceae	*Artemisia absinthium* L.	Aerial parts	[130]
*Arabidopsis thaliana* (L.) Heynh.	Juncaceae	*Juncus compressus* Jacq.	n.a.	[101]

* n.a.: not available.

### 4.3. Bioassays for Determining Herbicidal Activity

Based on the average pesticide consumption of the EU-27 Member States during the period of 2010–2019, herbicides represent more than 30% of all pesticides used in the EU [166], whereas worldwide, herbicides account for 50% of all pesticides used, of which >75% are used in developed countries [13]. The reduction in herbicide use premises the adoption of suitable, alternative weed management strategies. However, farmers tend to focus on the short-term economic benefits, whereas the agroecological benefits of herbicide reduction are long-term oriented. In contrast to the use of synthetic herbicides, bioherbicides are an ecologically sustainable alternative that is a priority in the EU. These eco-friendly herbicides can be subdivided into microbial bioherbicides and bio-derived (biochemical) bioherbicides. Microbial bioherbicides are made of bacteria, fungi, or viruses, either in their active form (liquid formulation) or in their dormant form (dry formulation). Natural molecules extracted, in most cases, from plants are the active ingredients of bio-derived bioherbicides. However, botanical products can be heterogeneous as a concenquence of the bioactive component mixture’s presence either from the same or from purposefully mixed botanical sources. Physical analytical methods, such as chromatography, are inadequate for this purpose, as they are often not sensitive enough to the chemical complexities found in crude botanical extracts. Most often, a desired biological response is owed to a mixture of bioactive plant components, and the relative proportions of single bioactive compounds may vary from batch to batch, whereas the bioactivity remains within tolerable limits. Thus, physical or chemical analysis of a single component in such mixtures is not completely satisfactory [167]. The isolation of plant allelopathic substances and the evaluation of their phytotoxic effects can lead to the discovery of new natural herbicides. For the above reasons, a decisive factor in the discovery of bioherbicides is the evaluation of the herbicidal activity of plant extracts by bioassays.

The herbicidal activity of plant extract evaluation can be estimated either at the laboratory scale using in vitro assays or in the field via pre- and postemergence assays. An in vitro assay evaluates the seed germination in Petri dishes. The inhibitory effects of the extract on weed seeds are determined by counting the germinated seeds (percent of germination), the root length of germinated seeds, the sprout length, etc. Firstly, it is crucial that the seed surface be sterilized to avoid possible inhibition of germination caused by fungal or bacterial toxins. The seeds are placed on a filter paper soaked by the extract [78] or covered by a soaked filter paper [61]. One concentration or multiple concentrations of the extract can be used during the assay [29]. The dishes are sealed with parafilm to avoid evaporation of the extract and incubated in certain temperature and photoperiod conditions. Variations of the method have been successfully employed to test extracts from various Mediterranean species against weeds such as *Melilotus officinalis* L., *Myosotis arvensis* (L.) Hill and Trigonella *besseriana* Ser. [61], and *Amaranthus retroflexus* L. and *Portulaca oleracea* L., *Stellaria media* (L.) Vill., and *Anagallis arvensis* [86]. The method can also be applied to germinating seedlings [120]. On the other hand, evaluation of the herbicidal activity can also be estimated in the field in pre- and postemergence assays. Morra et al. [66] evaluated the activity of *Sinapis alba* extract to the seeds of *Amaranthus powellii* and *Setaria viridis*. In preemergence assays, the solution of the extract is applied to the surface of the pot, whereas in postemergence assays, the extract either is sprayed or watered [117]. In preemergence assays, the emerged live seedlings, the plant height, and the dry weight are recorded, whereas in postemergence assays, the live plants per pot, the plant height, and the dry weight are determined [66].

### 4.4. Current and Future Research Trends in Biological Assays

Currently, classic in vitro microbiological methods, such as the agar dilution and disc diffusion methods, constitute the most common assays used for testing the antimicrobial activity of plant extracts [143]. These methods are generally preferred due to their low-cost design and simple execution, as well as their easily detectable and interpretable results. They provide apt evidence of bioactivity on a laboratory scale before testing promising extracts or metabolites on a larger scale in experimental plots in the field. These methods have been widely used for decades and will continue to be the preferred methods for initial bioactivity screening of compounds. However, there are certain shortcomings in their use, mostly related to their inefficiency with evaluating important parameters of the interaction between extracts and treated plants, as well as other biotic or abiotic elements of the environment. Typical in vitro antimicrobial assays usually fail to evaluate the toxic effects of biopesticides on non-target organisms and their residual persistence or degradation rate and instead simply report on the observed effect [71,76,78,89,106,116,117]. More sophisticated in situ assays can overcome these deficiencies and provide information on these critical parameters. For these reasons, they are constantly gaining ground with such bioassays. However, these assays are more complex to set and execute since they require significantly more resources and time for experimentation. They are also much more difficult to standardize compared to in vitro assays and are prone to serious experimental setbacks [45,54].

Similar issues arise for in vitro insecticidal and herbicidal assays. In these cases, there are also specific methods that are preferred by most researchers due to their simplicity (i.e., topical application or residual contact for insecticidal tests). In situ assays are slowly becoming more popular but may face additional limitations compared to antimicrobial assays—for instance, due to the mobility of flying insects [66,80,117]. The lack of standardized methods is often critical, especially in insecticidal or repellency assays where a variety of arenas is being used, with different general setups, dimensions, materials, etc. [44,70,72,107]. The adaptation of more standardized in situ techniques, such as olfactometers for repellency tests, will facilitate the reproducibility of the results of such bioassays. It will also improve the design of similar tests and the evaluation procedure for other extracts or compounds.

**Table 5:** Recent studies on bacterial, antiviral, and nematicidal activity of Mediterranean plant extracts/essential oils.

Control	Target Tested	Family	Plant	Parts Used for Extraction	References
Bacteria	*Clavibacter michiganensis*	Asteraceae	*Achillea ptarmica* L.	Aerial parts	[84]
			*Achillea millefolium* L.	Aerial parts	
			*Arctium lappa* L.	Aerial parts	
			*Bidens tripartite* L.	Aerial parts	
			*Carduus acanthoides* L.	Aerial parts	
			*Carduus nutans* subsp. *leiophyllus* (Petrović) Stoj. & Stef.	Aerial parts	
			*Centaurea cyanus* L.	Aerial parts	
			*Centaurea jacea* L.	Aerial parts	
			*Centaurea scabiosa* L.	Aerial parts	
			*Cirsium arvense* (L.) Scop.	Aerial parts	
			*Echinops ritro* L.	Aerial parts	
			*Gnaphalium uliginosum* L.	Aerial parts	
			*Pentanema britannica* (L.) D. Gut. Larr., Santos-Vicente, Anderb., E.Rico & M.M.Mart.Ort.	Aerial parts	
			*Sonchus arvensis* L.	Aerial parts	
			*Tripleurospermum inodorum* (L.) Sch. Bip.	Aerial parts	
		Compositae	*Leontodon hispidus* L.	Aerial parts	
			*Silybum marianum* (L.) Gaertn.	Aerial parts	
	*Pectobacterium carotovorum*	Apiaceae	*Carum carvi* L.	Seeds	[45]
		Asteraceae	*Achillea millefolium* L.	Stems, leaves, and flowers	
		Asteraceae	*Taraxacum officinale* F.H. Wigg. subsp. *officinale*	Leaves and stems	
		Cannabaceae	*Humulus lupulus* L.	Inflorescences	
		Clusiaceae	*Hypericum perforatum* L.	Root	
		Eqoisetaceae	*Equisetum arvense* L.	Leaves and stems	
		Lamiaceae	*Lavandula angustifolia* Mill.	Flower buds	
			*Mentha piperita* L.	Leaves, stems	
			*Rosmarinus officinalis* L.	Leaves, stems	
			*Salvia officinalis* L.	Stems	
			*Satureja hortensis* L.	Leaves and stems	
			*Thymus leucotrichus* Halácsy	Seeds	
		Poaceae	*Echinochloa crus-galli* (L.) P. Beauv.	Leaves	[117]
		Poaceae	*Elymus repens* (L.)	Leaves and stems	[45]
		Polygonaceae	*Polygonum aviculare* L.	Leaves and stems	
		Polygonaceae	*Polygonum bistorta* L. Samp.	Leaves and stems	
		Ranunculaceae	*Nigella sativa* L.	Seeds	
		Urticaceae	*Urtica dioica* L.	Stems	
Virus	Tobacco Mosaic Virus	Solanaceae	*Hyoscyamus niger* L.	Seeds	[121]
Clitellata	*Eisenia fetida*	Asteraceae	*Artemisia absinthium* L. (var. Candial)	n.a. *	[165]
		Lamiaceae	*Origanum syriacum* L. subsp. *syriacum*	Leaves	[25]
	*Panagrolaimus rigidus*	Lamiaceae	*Nepeta curviflora* Webb & Berthel.	Flowering tops, seeds, and leaves	[109]
		Lamiaceae	*Nepeta nuda* L. ssp. *pubescens*	Flowering tops, seeds, and leaves	
Nematodes	*Meloidogyne incognita*	Urticaceae	*Urtica dioica* L.	Whole plant	[122]
	*Meloidogyne javanica*	Lamiaceae	*Satureja montana* L.	Leaves and flowers	[114]

* n.a.: not available.

## 5. Toxicity and Safety Concerns

In general, biopesticides have nontoxic ways of action and are more selective in their targets than synthetic chemical pesticides [13]. However, some compounds in high doses may provoke toxic effects in nontarget organisms. Several suggestions, guidelines, regulations, and directives about biopesticides and their registration process have been published by agencies worldwide. For example, Regulation (EC) No. 1107/2009 requires analysis of impurities from plant protection products by toxicological and environmental testing [168]. Moreover, the FAO (2017), with its guidelines for the registration of microbial, botanical, and semiochemical pest control agents for plant protection and public health uses, requires (if previous assessments are not available or sufficient) acute and/or longer-term studies [3]. The US EPA (2012) indicates that the dose limit for most pesticides is 25 μg of active ingredient per *Apis mellifera* L. honeybee [169]. 

In Table 6, indicative recent studies that conducted toxicity assessments of plant extracts/essential oils are compiled. Recently, Di Lecce et al. [78] studied the potential toxic effects of extracts from seven plant species. The authors observed the toxicity of some extracts towards hepatocarcinoma Huh7 and the cytotoxicity towards ileocecal colorectal adenocarcinoma HCT-8 cell lines. In addition, phytotoxicity assays were conducted, and it was revealed that some extracts inhibited tomato rootlet elongation and seed cress germination. In 2017, Umpiérrez et al., investigating extracts from *Artemisia absinthium* L. and *Eupatorium buniifolium* and their effects on different seeds and insects, noticed that both extracts affected tomato seeds’ relative germination, germination rates, and the length that roots reach when exposed to high doses [130]. When an acute toxicity test was conducted on honeybees, the LD_50_ values were higher than those that the US EPA (2012) indicates, meaning that both extracts were considered safe [169]. Furthermore, exposure of 3% (*v*/*v*) of *Eupatorium buniifolium* extract to the Cetia variety led to acute toxic effects on whiteflies. On the other hand, 4.5% (*v*/*v*) led to necrotic effects on the vegetative parts of the plant. Cell cultures, *Caenorhabditis elegans*, and hen’s eggs were exposed to rosemary, *Citrus* and *Eucalyptus* oils by Lanzerstorfer et al. [170]. A dose-dependent decrease in cell viability with an IC_50_ ranging between 0.08 and 0.17% (*v*/*v*) was observed. The mean LC_50_ value for all oils of *Caenorhabditis elegans* was 0.42% (*v*/*v*). Moreover, the oils led to mucous membrane irritation signs.

Based on the available literature data and the legislation on biopesticides, the importance of the evaluation of potential hazards that plant extracts and essential oils might pose to nontarget plants, insects, etc., is highlighted. Although in most cases the toxic effects are dose dependent, occasionally even at low concentrations they can cause adverse effects. Especially for plant extracts, the potential toxic effects of the solvent used as a carrier should also be considered.

## 6. Conclusions and Future Perspectives

Botanical pesticides have long been touted as attractive alternatives to synthetic chemical pesticides for pest management, as they reputedly pose little threat to the environment and to human health. They are assumed to be harmless for farmers, easily biodegradable, and less toxic to non-target organisms. The growing number of studies that have recently investigated Mediterranean plants and that are reviewed in this study (Table 1, Table 2, Table 3, Table 4 and Table 5) demonstrate their effectiveness and suitability as sustainable and environmentally friendly biopesticides. Their various and novel modes of action are attributed to the specific phytochemical compositions (Table 1), which are affected by several factors, such as plant species or cultivar, geographical origin, environmental conditions, and agricultural practices. In addition, the choice of extraction method was found to be of primary importance for the quantity and quality of phytochemicals (Table 1). Based on the literature data presented in Table 1, the most used methods are the conventional extraction methods of hydrodistillation, Soxhlet extraction or hot continuous extraction, and maceration, which possess some limitations. To overcome the limitations of conventional extraction methods, new green methods can also be suggested (Figure 1), considering the potential impact on the environment. These methods could be adopted and developed by focusing on less hazardous solvents, the reduction of energy consumption, and safety, in terms of circular economy.

It is very important for biopesticides and related products to be evaluated in a more biological, ecological, and economic context. Up to now, most bioassays have been conducted at the laboratory scale, as can be seen in Table 6. However, the few data of experiments in the field area significantly limit the commercialization of biopesticide products. Further investigation is required to reassure the effectiveness of biopesticides in real conditions, developing suitable formulations that protect the compounds and release them slowly to the environment.

Consequently, several challenges need to be addressed before commercialization of biopesticides. A significant challenge for biopesticide development is the increase in their effectiveness. One reason for restricted use of biopesticides by farmers is the high degradation rate owing to their volatility, which leads to multiple treatments and increased production cost. To ensure their effectiveness and stability, the formulation of biopesticides must be improved, with minimum influence of external environmental factors such as temperature. Nanotechnology is a promising science with huge potential to provide novel approaches and solutions in the biopesticide sector and enhance the stability and efficiency of biopesticide nano-formulations. This means that it is necessary to intensify biopesticide development and that researchers must focus on the production, formulation, and application of them.

In addition, a key factor to determining the suitability of biopesticides is regulatory approval. In general, there is a strict framework for authorization that delays the promotion of products. As biopesticides are a low-risk and eco-friendly product, they must not be evaluated in the same way as chemical pesticides. Thus, the approval of an application for a biopesticide by the authorities should be a simple, rapid, less expensive procedure, different from that of chemical pesticides, to facilitate the registration of biopesticide products. 

Considering the increase in population and simultaneously the increasing demand for food, the use of biopesticides is an ecological solution to crop protection. Nevertheless, measures should be taken in order for the cultivation of the raw material (plants) to produce biopesticides to not affect global nutritional sufficiency and to not put pressure on food production. Moreover, agricultural waste as a source of active compounds could be a promising, circular, and cheap raw material for biopesticides. In general, farmers and society should benefit from the use of biopesticides. Regarding farmers, the effectiveness and reliability of biopesticides compared to synthetic chemical pesticides are the most important criteria for their acceptability. Emphasis should be placed on the benefits of biopesticide use. This could be supported by publicly funded programs, as well as pesticide firms, in order to inform farmers about the availability, use, and advantages of adopting biopesticides. This is in line with the Farm to Fork Strategy, which aims to ensure food safety in an environmentally sustainable manner and simultaneously maximize environmental, health, and social benefits.

## Figures and Tables

**Table 2:** Recent studies on antimicrobial activity of Mediterranean plant extracts/essential oils.

Fungus Tested	Family	*Plant*	References
*Alternaria alternata*	Lamiaceae	*Lavandula canariensis* Mill.	[105]
	Rutaceae	*Ruta chalepensis* L.	
*Alternaria solani*	Lamiaceae	*Mentha piperita* L.	[106]
		*Rosmarinus officinalis* L.	
	Poaceae	*Echinochloa crus-galli* (L.) P. Beauv.	[117]
*Alternaria* spp.	Lamiaceae	*Thymus leucotrichus* Halácsy	[60]
		*Hyssopus officinalis* L.	
	Cupressaceae	*Juniperus communis* L.	
*Botrytis cinerea*	Cupressaceae	*Juniperus communis* L.	[59]
		*Juniperus oxycedrus* L.	
		*Juniperus communis* L. var. *saxatilis* Pall.	
	Lamiaceae	*Lavandula canariensis* Mill.	[105]
	Rutaceae	*Ruta chalepensis* L.	
	Lauraceae	*Laurus nobilis* L.	[116]
*Cercospora kikuchii*	Lamiaceae	*Lavandula dentata* L.	[104]
*Cercospora sojina*	Lamiaceae	*Lavandula dentata* L.	[104]
*Colletotrichum* spp.	Cupressaceae	*Juniperus communis* L.	[59]
		*Juniperus oxycedrus* L.	
		*Juniperus communis* L. var. *saxatilis* Pall.	
*Cylindrocarpon pauciseptatum*	Cupressaceae	*Juniperus communis* L.	[59]
		*Juniperus oxycedrus* L.	
		*Juniperus communis* L. *var. saxatilis* Pall.	
*Fusarium culmorum*	Salicaceae	*Populus tremula* L.	[120]
*Fusarium oxysporum* f. sp. *lycopersici*.	Lamiaceae	*Mentha piperita* L.	[106]
	Lamiaceae	*Rosmarinus officinalis* L.	
*Fusarium oxysporum*	Lamiaceae	*Lavandula canariensis* Mill.	[105]
	Rutaceae	*Ruta chalepensis* L.	
	Lamiaceae	*Mentha piperita* L.	[106]
		*Rosmarinus officinalis* L.	
*Fusarium* spp.	Cupressaceae	*Juniperus communis* L. var. *saxatilis* Pall.	[59]
		*Juniperus oxycedrus* L.	
		*Juniperus communis* L.	
*Geotrichum candidum* var. *citri-aurantii*	Cistaceae	*Cistus albidus* L.	[54]
		*Cistus creticus* L.	
		*Cistus crispus* L.	
		*Cistus ladanifer* L.	
		*Cistus laurifolius* L.	
		*Cistus monspeliensis* L.	
		*Cistus populifolius* L.	
		*Cistus salviifolius* L.	
*Mycosphaerella graminicola*	Lamiaceae	*Thymus leucotrichus* Halácsy	[30]
*Penicillium allii*	Lamiaceae	*Origanum vulgare* L.	[111]
*Phoma exigua*	Lamiaceae	*Rosmarinus officinalis* L.	[45]
		*Salvia officinalis* L.	
		*Satureja hortensis* L.	
		*Thymus leucotrichus* Halácsy L.	
	Poaceae	*Elytrigia repens* (L.) Nevski	
	Polygonaceae	*Polygonum aviculare* L.	
		*Persicaria bistorta* (L.) Samp.	
	Ranunculaceae	*Nigella sativa* L.	
	Urticaceae	*Urtica dioica* L.	
*Pythium ultimum*	Lamiaceae	*Rosmarinus officinalis* L.	[106]
		*Mentha piperita* L.	
*Rhizoctonia solani*	Cupressaceae	*Juniperus communis* L.	[59]
		*Juniperus oxycedrus* L.	
		*Juniperus communis* L. var. *saxatilis* Pall.	
	Lamiaceae	*Mentha piperita* L.	[106]
		*Rosmarinus officinalis* L.	
*Sclerotinia sclerotiorum*	Apiaceae	*Cuminum cyminum* L.	[76]
*Septoria glycines*	Lamiaceae	*Lavandula dentata* L.	[104]
*Verticillium dahliae*	Anacardiaceae	*Pistacia lentiscus* L.	[71]
	Apocynaceae	*Nerium oleander* L.	
	Araliaceae	*Hedera helix* L.	
	Asteraceae	*Dittrichia viscosa* (L.) Greuter	
	Brassicaceae	*Brassica rapa* L.	
		*Diplotaxis erucoides* (L.) DC.	
		*Diplotaxis virgata* (Cav.) DC.	
		*Hirschfeldia incana* (L.) Lagr.-Foss.	
	Cistaceae	*Cistus albidus* L.	
		*Cistus ladanifer* L.	
		*Cistus laurifolius* L.	
	Cupressaceae	*Juniperus communis* L.	
	Fagaceae	*Castanea sativa* Mill.	
	Junglandaceae	*Juglans regia* L.	
	Lamiaceae	*Marrubium vulgare* L.	
		*Mentha x verticillata* L.	
		*Origanum vulgare* L.	
		*Rosmarinus officinalis* L.	
		*Salvia officinalis* L.	
		*Thymus leucotrichus* Halácsy	
		*Laurus nobilis* L.	
	Oleaceae	*Olea europaea* cv. Lechín de Sevilla	
		*Olea europea* cv. Arbequina	
		*Olea europea* cv. Cornicabra	
		*Olea europea* cv. Empeltre	
		*Olea europea* cv. Frantoio	
		*Olea europea* cv. Picual	
	Papaveraceae	*Papaver rhoeas* L.	
	Pinaceae	*Pinus pinea* L.	
	Urticaceae	*Urtica* sp.	
	Viburnaceae	*Sambucus nigra* L.	
*Zymoseptoria tritici*	Cannabaceae	*Humulus lupulus* L.	[95]
*Alternaria alternata*/*Alternaria solani*/*Alternaria tenuissima*/*Colletotrichum coccodes*/*Fusarium oxysporum*/*Fusarium sambucinum*/*Rhizoctonia solani*/*Streptomyces scabiei*	Apiaceae	*Carum carvi* L.	[45]
	Lamiaceae	*Thymus leucotrichus* Halácsy L.	
	Asteraceae	*Achillea millefolium* L.	
		*Taraxacum officinale* (L.) Weber ex F.H.Wigg	
	Cannabaceae	*Humulus lupulus* L.	
	Clusiaceae	*Hypericum perforatum* L.	
	Eqoisetaceae	*Equisetum arvense* L.	
	Lamiaceae	*Salvia officinalis* L.	
		*Mentha piperita* L.	
		*Rosmarinus officinalis* L.	
		*Lavandula angustifolia* Mill.	
		*Satureja hortensis* L.	
	Poaceae	*Elytrigia repens* (L.) Nevski	
	Polygonaceae	*Polygonum aviculare* L.	
		*Persicaria bistorta* (L.) Samp.	
	Ranunculaceae	*Nigella sativa* L.	
	Urticaceae	*Urtica dioica* L.	

**Table 6:** Recent studies on toxicity assessments of plant extracts/essential oils.

Extract	Method/Organism	References
*Prasium majus* L.*, Glaucium flavum* Crantz*, Daucus lopadusanus* Tineo*, Periploca angustifolia* Labill*, Echinops spinosissimus* Turra*, Hypericum aegypticum* L.	*Solanum lycopersicum* L.	[78]
*Prasium majus* L.*, Glaucium flavum* Crantz*, Daucus lopadusanus* Tineo*, Periploca angustifolia* Labill*, Echinops spinosissimus* Turra*, Hypericum aegypticum* L.	MTT-based colorimetric assay/hepatocarcinoma Huh7 cell lines/ideocecal colorectal adenocarcinoma HCT-8 cell lines	
*Artemisia absinthium* L.	*Solanum lycopersicum* L. (Mirella and Cetia seeds)	[130]
	EPA OCSPP 850.3020 and complete exposure test/*Apis mellifera* L.	
*Eupatorium buniifolium* Hook. & Arn.	*Solanum lycopersicum* L. (Mirella and Cetia seeds)	
	EPA OCSPP 850.3020 and complete exposure test/*Apis mellifera* L.	
	Greenhouse assay/*Solanum lycopersicum* L. (Cetia seeds) and whitflies	
Rosemary oil, citrus oil, eucalyptus oil	Resazurin-based in vitro toxicology assay/HeLa cell lines/Caco-2 cell lines/STF1 cell lines	[170]
	*Caenorhabditis elegans*	
	Hen’s eggs (Lohmann classic brown chicken)	
